# Reciprocal regulation of enterococcal cephalosporin resistance by products of the autoregulated *yvcJ-glmR-yvcL* operon enhances fitness during cephalosporin exposure

**DOI:** 10.1371/journal.pgen.1011215

**Published:** 2024-03-21

**Authors:** Dušanka Djorić, Samantha N. Atkinson, Christopher J. Kristich

**Affiliations:** 1 Department of Microbiology and Immunology, Medical College of Wisconsin, Milwaukee, Wisconsin, United States of America; 2 Center for Microbiome Research, Medical College of Wisconsin, Milwaukee, Wisconsin, United States of America; The University of Texas Health Science Center at Houston, UNITED STATES

## Abstract

Enterococci are commensal members of the gastrointestinal tract and also major nosocomial pathogens. They possess both intrinsic and acquired resistance to many antibiotics, including intrinsic resistance to cephalosporins that target bacterial cell wall synthesis. These antimicrobial resistance traits make enterococcal infections challenging to treat. Moreover, prior therapy with antibiotics, including broad-spectrum cephalosporins, promotes enterococcal proliferation in the gut, resulting in dissemination to other sites of the body and subsequent infection. As a result, a better understanding of mechanisms of cephalosporin resistance is needed to enable development of new therapies to treat or prevent enterococcal infections. We previously reported that flow of metabolites through the peptidoglycan biosynthesis pathway is one determinant of enterococcal cephalosporin resistance. One factor that has been implicated in regulating flow of metabolites into cell wall biosynthesis pathways of other Gram-positive bacteria is GlmR. In enterococci, GlmR is encoded as the middle gene of a predicted 3-gene operon along with YvcJ and YvcL, whose functions are poorly understood. Here we use genetics and biochemistry to investigate the function of the enterococcal *yvcJ-glmR-yvcL* gene cluster. Our results reveal that YvcL is a DNA-binding protein that regulates expression of the *yvcJ-glmR-yvcL* operon in response to cell wall stress. YvcJ and GlmR bind UDP-GlcNAc and reciprocally regulate cephalosporin resistance in *E*. *faecalis*, and binding of UDP-GlcNAc by YvcJ appears essential for its activity. Reciprocal regulation by YvcJ/GlmR is essential for fitness during exposure to cephalosporin stress. Additionally, our results indicate that enterococcal GlmR likely acts by a different mechanism than the previously studied GlmR of *Bacillus subtilis*, suggesting that the YvcJ/GlmR regulatory module has evolved unique targets in different species of bacteria.

## Author summary

Enterococci are commensal members of the gastrointestinal tract and also major nosocomial pathogens. They possess both intrinsic and acquired resistance to many antibiotics, including intrinsic resistance to cephalosporins that target bacterial cell wall synthesis, rendering enterococcal infections challenging to treat. Moreover, prior therapy with antibiotics, including cephalosporins, is a major risk factor for subsequent acquisition of an enterococcal infection. Hence, a better understanding of mechanisms of cephalosporin resistance is needed to enable development of new therapies to treat or prevent enterococcal infections. We investigated the function of the well-conserved *yvcJ-glmR-yvcL* gene cluster in enterococcal cephalosporin resistance, revealing that YvcJ and GlmR reciprocally regulate cephalosporin resistance in *E*. *faecalis*, and this regulation is essential for fitness during exposure to cephalosporin stress. Additionally, our results indicate that enterococcal GlmR likely acts by a different mechanism than the previously studied GlmR of *Bacillus subtilis*, suggesting that the YvcJ/GlmR regulatory module has evolved unique targets in different species of bacteria.

## Introduction

Enterococci are commensal members of the gastrointestinal tract as well as major nosocomial pathogens that possess intrinsic resistance to many antibiotics, including cephalosporins [1–6]. Cephalosporins inhibit cell wall biosynthesis by acylating the active-site serine of penicillin-binding proteins, thereby preventing cross-linking of the peptidoglycan (PG) and resulting in cell death [7–9], but enterococci possess as-yet incompletely characterized resistance mechanisms that allow them to overcome cephalosporin challenge. *Enterococcus faecalis* and *Enterococcus faecium* account for most hospital-acquired infections [5,6,10] and these infections are often associated with significant morbidity and mortality [6,11]. Enterococcal strains responsible for hospital-acquired infection often have additional resistance traits rendering them multi-drug resistant and thus challenging to treat [3,12,13]. Additionally, prior therapy with antibiotics, such as broad-spectrum cephalosporins, promotes enterococcal proliferation in the gut, resulting in dissemination to other sites of the body and infection [14–19]. Molecular mechanisms responsible for intrinsic cephalosporin resistance have not been completely elucidated, and a better understanding of these mechanisms is needed to enable development of new therapies to treat or prevent enterococcal infections.

We previously showed that flow of metabolites through the PG biosynthesis pathway serves as an important driver of enterococcal cephalosporin resistance [20]. One protein that has been studied in other Gram-positive bacteria and that directs metabolites into the cell wall synthesis pathway in *Bacillus subtilis* is GlmR (also known as YvcK and CuvA) [21–27]. Mutants lacking *glmR* are sensitive to certain cell-wall acting antibiotics, and–in *B*. *subtilis* at least–are unable to grow on gluconeogenic carbon sources [22,24,26,27]. *B*. *subtilis* GlmR boosts the activity of GlmS, which converts fructose-6-P to glucosamine-6-P, thereby diverting metabolites from glycolysis into the cell wall synthesis pathway. Thus, GlmR action serves to enhance cell wall synthesis [27]. Another connection between GlmR and cell wall synthesis also exists: recently, GlmR of *Listeria monocytogenes* was reported to act as a uridyltransferase, catalyzing conversion of GlcNAc-1P + UTP to UDP-GlcNAc, a key metabolic intermediate in the synthesis of multiple cell wall components, including PG [28]. Given these findings with GlmR homologs from other Gram-positive bacteria, we sought to determine if GlmR might play a role in regulation of enterococcal intrinsic cephalosporin resistance. Our results revealed that GlmR is indeed required for cephalosporin resistance in enterococci.

GlmR is encoded as the middle gene of a predicted 3-gene operon along with YvcJ and YvcL, whose functions are poorly understood. Hypothesizing that products from the adjacent genes might be functionally related to GlmR, we used genetics and biochemistry to also investigate the function of YvcJ and YvcL. We found that YvcL is a DNA-binding protein that regulates expression of the *yvcJ-glmR-yvcL* operon in response to cell wall stress. YvcJ and GlmR bind UDP-GlcNAc and reciprocally regulate cephalosporin resistance in *E*. *faecalis*. Though the specific molecular targets for regulation by GlmR and YvcJ in *E*. *faecalis* remain unknown, binding of UDP-GlcNAc by YvcJ appears essential for its activity, and reciprocal regulation by YvcJ/GlmR is essential for fitness during exposure to cephalosporin stress. Additionally, our results indicate that enterococcal GlmR likely acts by a different mechanism than GlmR of *B*. *subtilis*, suggesting that the YvcJ/GlmR regulatory module has evolved unique targets in different species of bacteria.

## Results

### GlmR is required for enterococcal cephalosporin resistance

In *B*. *subtilis* and *L*. *monocytogenes*, mutants lacking *glmR* exhibit defects in cell wall homeostasis and reduced resistance to cephalosporins. To determine if enterococcal *glmR* influences cephalosporin resistance, we created in-frame deletions of *glmR* in two evolutionarily divergent lineages of *E*. *faecalis* (OG1 [locus *OG1RF_10501*] and CK221 [locus *EF0767*]), as well as an *E*. *faecium* strain (1141733; locus *EFSG_RS09475*), and assessed resistance of the resulting mutants to a panel of antibiotics (Table 1). Deletion of *glmR* in all three enterococcal lineages resulted in reduced resistance to cephalosporins, indicating that GlmR promotes enterococcal cephalosporin resistance. In *E*. *faecium*, deletion of *glmR* also resulted in a notable reduction in resistance to ampicillin. Resistance to other antibiotics, some of which also target cell wall synthesis, remained largely unchanged, suggesting that the defect in cephalosporin resistance was not due to a general loss of stress resistance. Supplementation of culture media with high concentrations of magnesium chloride can sometimes suppress defects in peptidoglycan synthesis of other Gram-positive bacteria, but this was not the case with the Δ*glmR* mutant (S1 Table). Complementation analysis was performed by expressing *glmR* from *E*. *faecalis* OG1 on a plasmid with a constitutive promoter in each of the three Δ*glmR* mutants, revealing that GlmR overexpression not only rescued the ceftriaxone resistance defect of each but also led to hyper-resistance (S2 Table), confirming that the Δ*glmR* mutations were responsible for the cephalosporin resistance defects, and suggesting a positive correlation between GlmR levels and cephalosporin resistance. Immunoblot analysis revealed that GlmR was indeed overexpressed from the constitutive promoter on this plasmid (S1 Fig). Moreover, these results indicate that the GlmR homologs from *E*. *faecalis* and *E*. *faecium* are functionally equivalent given the cross-species complementation observed, which we further confirmed by demonstrating that expression of the *E*. *faecium glmR* homolog in the *E*. *faecalis* OG1 Δ*glmR* mutant also successfully cross-complemented (S3 Table). To determine if cephalosporin resistance is correlated with GlmR levels, we used an inducible promoter to regulate GlmR expression, revealing that inducible expression of GlmR (S2 Fig) resulted in a dose-dependent increase in ceftriaxone resistance (Table 2). Immunoblotting revealed that GlmR levels when expressed from the inducible promoter did not achieve same level observed with the constitutive promoter, likely explaining why expression from the inducible promoter did not result in hyper-resistance (S3 Fig). Taken together, the data indicate that GlmR from both *E*. *faecalis* and *E*. *faecium* drives enterococcal cephalosporin resistance.

**Table 1:** Antimicrobial resistance of Δ*glmR* mutants.

	MIC^a^ (μg/ml)					
	WT_OG1_	Δ*glmR*_OG1_	WT_CK221_	Δ*glmR*_CK221_	WT_*E*. *faecium*_	Δ*glmR*_*E*. *faecium*_
*Cell wall targets*						
Ceftriaxone	64	8	512	8	64	1
Cefuroxime	128	8	512	4	512	16
Fosfomycin	32	32	64	32	64	64
Bacitracin	64	64	16	16	64	64
D-cycloserine	128	128	128	128	64	64
Ampicillin	1	0.5	0.5	0.5	1	0.125
*Other targets*						
Chloramphenicol	8	8	8	8	4	4
Norfloxacin	4	4	1	1	2	2
Erythromycin	1	1	1	1	2	2

^a^Median minimal inhibitory concentrations (MICs) determined in MH broth after a 24 h incubation at 37°C, from a minimum of three independent experiments. Strains were: wild-type (WT) *E*. *faecalis* (OG1 or CK221); wild-type *E*. *faecium* (1,141,733); Δ*glmR*_OG1_, DDJ245; Δ*glmR*_CK221_, DDJ248, Δ*glmR*_*E*. *faecium*_, DDJ262.

**Table 2:** Inducible expression of GlmR results in dose-dependent increases in ceftriaxone resistance.

	MIC^a^_ceftx_ (μg/ml)				
[NaNO_3_] (mM)	0	0.5	1	5	10
Strain^b^					
Wild-type (vector)	128	128	128	128	128
Wild-type (P_nisA_-*glmR*)	64	128	128	256	256
Δ*glmR* (vector)	8	8	8	8	8
Δ*glmR* (P_nisA_-*glmR*)	32	64	64	128	256
Δ*glmR* (P_nisA_-*glmR*_N206A_)	16	nd^c^	nd^c^	32	32
Δ*glmR* (P_nisA_-*glmR*_D42A D43A N206A_)	8	nd^c^	nd^c^	8	8
Δ*glmR* (P_nisA_-*glmR*_D42A D43A_)	8	nd^c^	nd^c^	8	8

^a^Median minimal inhibitory concentrations for ceftriaxone (MIC_ceftx_) determined in MH broth (supplemented with 10 μg/ml erythromycin and indicated nitrate concentrations) after 24 h at 37°C, from a minimum of three independent experiments.

^b^Strains were: Wild-type, *E*. *faecalis* OG1; Δ*glmR*, DDJ245. Plasmids were: vector, pJLL286; P_nisA_-*glmR*, pDDJ262; P_nisA_-*glmR*_N206A_, pDDJ303; P_nisA_-*glmR*_D42A D43A N206A_, pDDJ306; P_nisA_-*glmR*_D42A D43A_, pDDJ307.

^c^nd, not determined.

### *E*. *faecalis* GlmR functions differently than *B*. *subtilis* GlmR

The *B*. *subtilis glmR* deletion mutant has been shown to have growth defects as well as increased susceptibility to certain antibiotics [22,24,27]. Additionally, *B*. *subtilis* GlmR has been implicated in regulation of central carbon metabolism due to the inability of a Δ*glmR* mutant to grow on gluconeogenic carbon sources, and the ability of GlmS upregulation to rescue some of the associated defects [22,27]. To determine if enterococcal GlmR functions in a similar way, we assessed growth of Δ*glmR* mutants in the three enterococcal lineages. No defects in exponential growth were observed in the gluconeogenic Mueller-Hinton broth (S4 Fig), in contrast to what is observed in *B*. *subtilis* (although the enterococcal Δ*glmR* mutants may exhibit a slightly enhanced lysis phenotype in stationary phase). Additionally, no growth defects were observed for the *E*. *faecalis* OG1 Δ*glmR* mutant cultured in a semi-defined medium using either glycolytic or gluconeogenic carbon sources, including ribose, pyruvate, glycerol, glucose, or N-acetylglucosamine (S5 Fig). Hence the most obvious metabolic defects of the *B*. *subtilis* Δ*glmR* mutant are not shared in enterococci.

In *B*. *subtilis*, provision of exogenous GlcNAc can bypass the Δ*glmR* metabolic defect, because GlcNAc can be imported and converted to glucosamine-6-P (the product of GlmS), thereby bypassing the GlmS-catalyzed step of the pathway [22,27]. Similarly, upregulation of GlmS can bypass the metabolic defect of the *B*. *subtilis* Δ*glmR* mutant. However, neither provision of exogenous GlcNAc (S4 Table) nor expression of GlmS from a constitutive promoter (S6 Fig and S5 Table) resulted in rescue of ceftriaxone resistance for the *E*. *faecalis* OG1 Δ*glmR* mutant, consistent with the hypothesis that *E*. *faecalis* GlmR influences cephalosporin resistance through regulatory mechanisms that are distinct from those used by *B*. *subtilis* GlmR to influence cell wall synthesis.

A recent study reported that GlmR from several Gram-positive species of bacteria possesses uridyltransferase activity, catalyzing synthesis of UDP-GlcNAc from GlcNAc-1-P and UTP [28]. GlmR was therefore proposed to serve as an “accessory” uridyltransferase to augment the activity of GlmU, the bifunctional acetyltransferase/uridyltransferase that is normally responsible for synthesis of UDP-GlcNAc in the cell. In that study, a variant of *L*. *monocytogenes* GlmR simultaneously containing 3 substitutions lost its uridyltransferase activity *in vitro* (the D40A D41A N198A mutant). To test if uridyltransferase activity is important for function of *E*. *faecalis* GlmR, we constructed GlmR variants with mutations at the equivalent residues (D42, D43, N206 of *E*. *faecalis* GlmR) in various combinations. The N206A mutant was expressed at levels similar to wild-type GlmR (S7A Fig) and exhibited reduced ability to promote cephalosporin resistance (Table 2) although it was able to support a modest increase in resistance, suggesting the N206A substitution was partially inactivating. In contrast, simultaneous substitution of D42 and D43 with Ala in *E*. *faecalis* GlmR (with or without N206A substitution in combination) completely impaired the ability of GlmR to promote cephalosporin resistance (Table 2) despite being expressed as well as wild-type GlmR (S7A Fig), suggesting that uridyltransferase activity of *E*. *faecalis* GlmR could be important for its function. To further test the hypothesis that inadequate uridyltransferase activity in the cell is responsible for the loss of cephalosporin resistance of the Δ*glmR* mutant, we overexpressed *E*. *faecalis* GlmU in the OG1 Δ*glmR* mutant. However, overexpression of GlmU (S6 Fig) did not enhance cephalosporin resistance of the Δ*glmR* mutant (S5 Table). This result leaves open the possibility that the defects associated with the D42A D43A substitutions in GlmR may not be directly related to uridyltransferase activity *per se*. However, our data is not sufficient to definitively conclude that uridyltransferase activity is–or is not–required for GlmR to promote cephalosporin resistance because we have not measured cellular UDP-GlcNAc levels in the various strains tested here.

**Fig 1:**
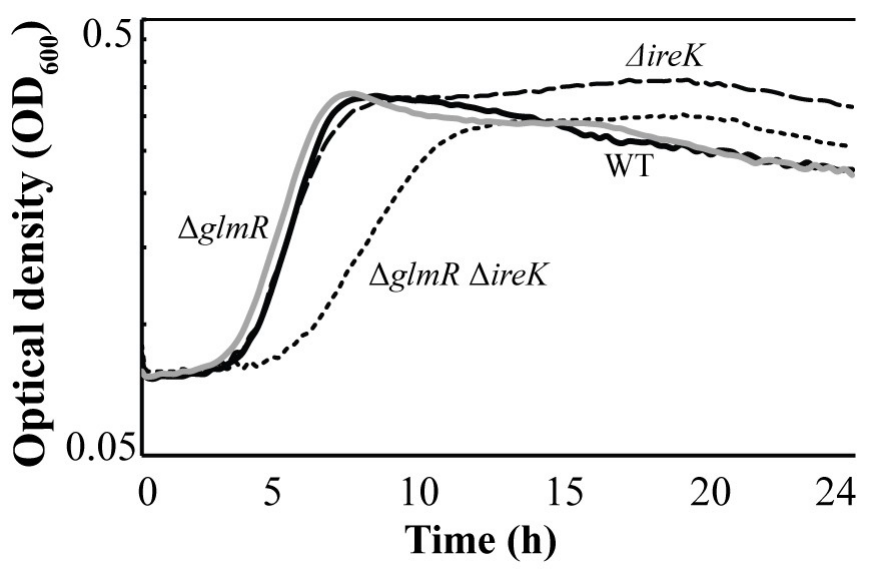
Δ*glmR* Δ*ireK* double mutant exhibits a growth defect in MH broth. Bacteria were grown in MH broth and culture density (OD_600_) measured using a Bioscreen C plate reader. Wild type (WT), full line; Δ*ireK* strain, dashed line; Δ*glmR* Δ*ireK* strain, dotted line; Δ*glmR*, gray line. Strains used: wild-type, OG1; Δ*ireK*, JL206; Δ*glmR* Δ*ireK*, DDJ332; Δ*glmR*, DDJ245.

GlmR homologs in *B*. *subtilis* and *L*. *monocytogenes* have been reported to be phosphorylated by a serine/threonine kinase of the PASTA kinase family [25,26]. The sites of phosphorylation identified in the *B*. *subtilis* and *L*. *monocytogenes* GlmR homologs are not conserved in *E*. *faecalis* GlmR, suggesting that *E*. *faecalis* GlmR may not be phosphorylated. Regardless, given that the only enterococcal PASTA kinase (IreK) is a key regulator of cephalosporin resistance, we tested whether IreK could phosphorylate *E*. *faecalis* GlmR *in vitro* using purified, recombinant proteins according to our previously described methods [29,30]. However, no phosphorylation of GlmR was observed. Therefore, we explored the relationship between IreK and GlmR using a genetic approach. To do so, we constructed a double mutant of *E*. *faecalis* OG1 lacking both *ireK* and *glmR* and assessed its growth and cephalosporin resistance. Unlike the otherwise isogenic single Δ*ireK* or Δ*glmR* mutants, the Δ*ireK* Δ*glmR* double mutant exhibited a notable growth defect (Fig 1) relative to wild-type and either of the single mutants. Additionally, the double mutant exhibited reduced resistance to ceftriaxone relative to either of the single mutants (Table 3). Finally, expression of GlmR from a plasmid in the Δ*ireK* mutant resulted in an 8-fold increase in ceftriaxone resistance. Collectively these results demonstrate that GlmR can act independently of IreK to influence enterococcal growth and cephalosporin resistance.

**Table 3:** Ceftriaxone resistance of mutants indicates GlmR and IreK act independently.

Strain^b^	MIC^a^_ceftx_ (μg/ml)
Wild-type	64
Δ*glmR*	8
Δ*ireK*	1
Δ*glmR* Δ*ireK*	0.5
Δ*ireK* (vector)	1
Δ*ireK* (P-*glmR*)	8

^a^Median minimal inhibitory concentrations for ceftriaxone (MIC_ceftx_) determined in MH broth (supplemented with 10 μg/ml chloramphenicol for plasmid maintenance when necessary) after 24 h incubation at 37°C, from a minimum of three independent experiments.

^b^Strains were: Δ*ireK*, JL206; Δ*glmR*, DDJ245; Δ*glmR* Δ*ireK*, DDJ322. Plasmids were: vector, pJRG9; P-*glmR*, pJLL238.

### *glmR* exists in an operon with *yvcJ* and *yvcL*

**Fig 2:**
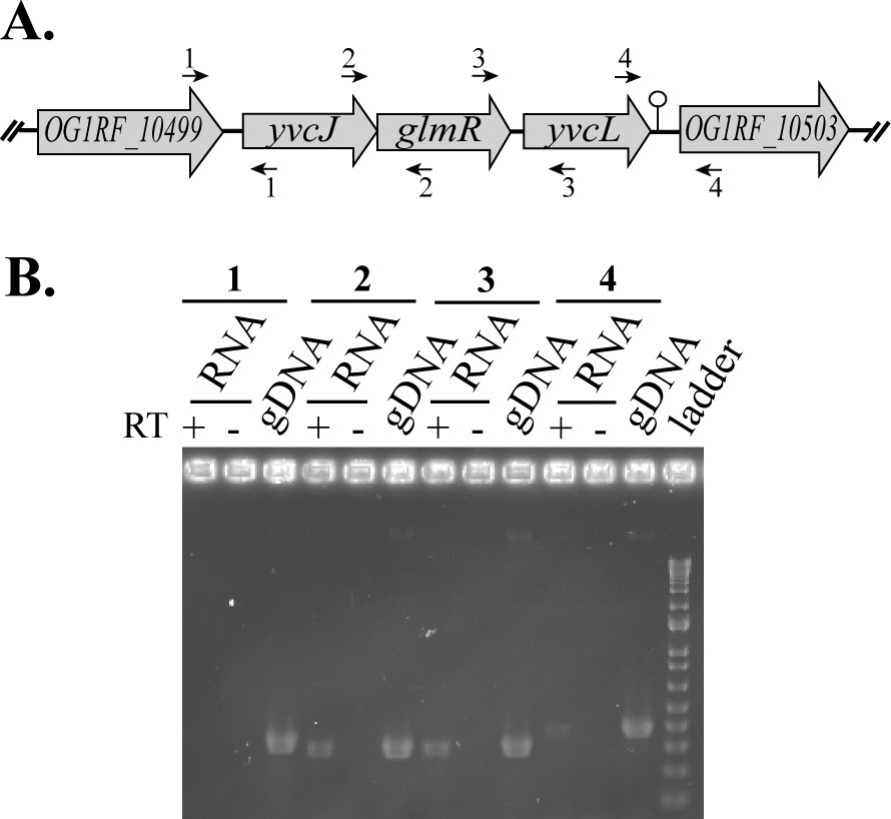
*glmR* is in an operon with *yvcJ* and *yvcL*. **A.** Schematic of the *glmR* genetic locus on the *E*. *faeca*lis chromosome (not to scale). A predicted terminator is depicted at the end of *yvcL*. **B.** Templates for PCR are indicated above the gel. PCR was performed with or without reverse transcriptase (RT) on RNA purified from exponentially growing OG1 (wild-type) in order to assess presence of specific transcripts using primer sets as depicted in (A). OG1RF genomic DNA (gDNA) was used as a control for the PCR.

The *glmR* gene overlaps the gene encoded immediately upstream (*yvcJ*; Fig 2A) by a few nucleotides. In *B*. *subtilis*, the YvcJ protein interacts with GlmR and is thought to regulate GlmR activity [31]. To examine conservation of the gene neighborhood around *glmR*, multigene BLAST [32] was used to analyze GlmR and its flanking genes, revealing the three-gene cluster (*yvcJ-glmR-yvcL*) to be highly conserved in Gram-positive bacteria (S8 Fig). Previously *yvcJ-glmR-yvcL* were predicted to be in an operon together flanked by a transcriptional terminator located downstream of *yvcL* [33,34]. To determine if these genes might be transcriptionally linked in *E*. *faecalis*, we used RT-PCR to examine the transcriptional organization of the *glmR* locus. We observed clear evidence for the presence of transcripts spanning the *yvcJ*-*glmR* intergenic region and the *glmR*-*yvcL* intergenic region, indicating that *yvcJ*, *glmR* and *yvcL* are likely co-transcribed from a common promoter (Fig 2B). A weak signal was observed for transcripts that spanned the *yvcL*-*OG1RF_10503* intergenic region, which could reflect low-level readthrough of a predicted transcriptional terminator [34] into OG1RF_10503. No transcripts were detected that spanned the *OG1RF_10499*-*yvcJ* intergenic region (Fig 2B). Hence, while it remains unclear if OG1RF_10503 is an authentic member of the operon, evolutionary conservation of the gene cluster and co-transcription of *yvcJ*, *glmR*, and *yvcL* suggested a functional relationship between them.

### YvcL regulates expression of *yvcJ* and *glmR*

**Fig 3:**
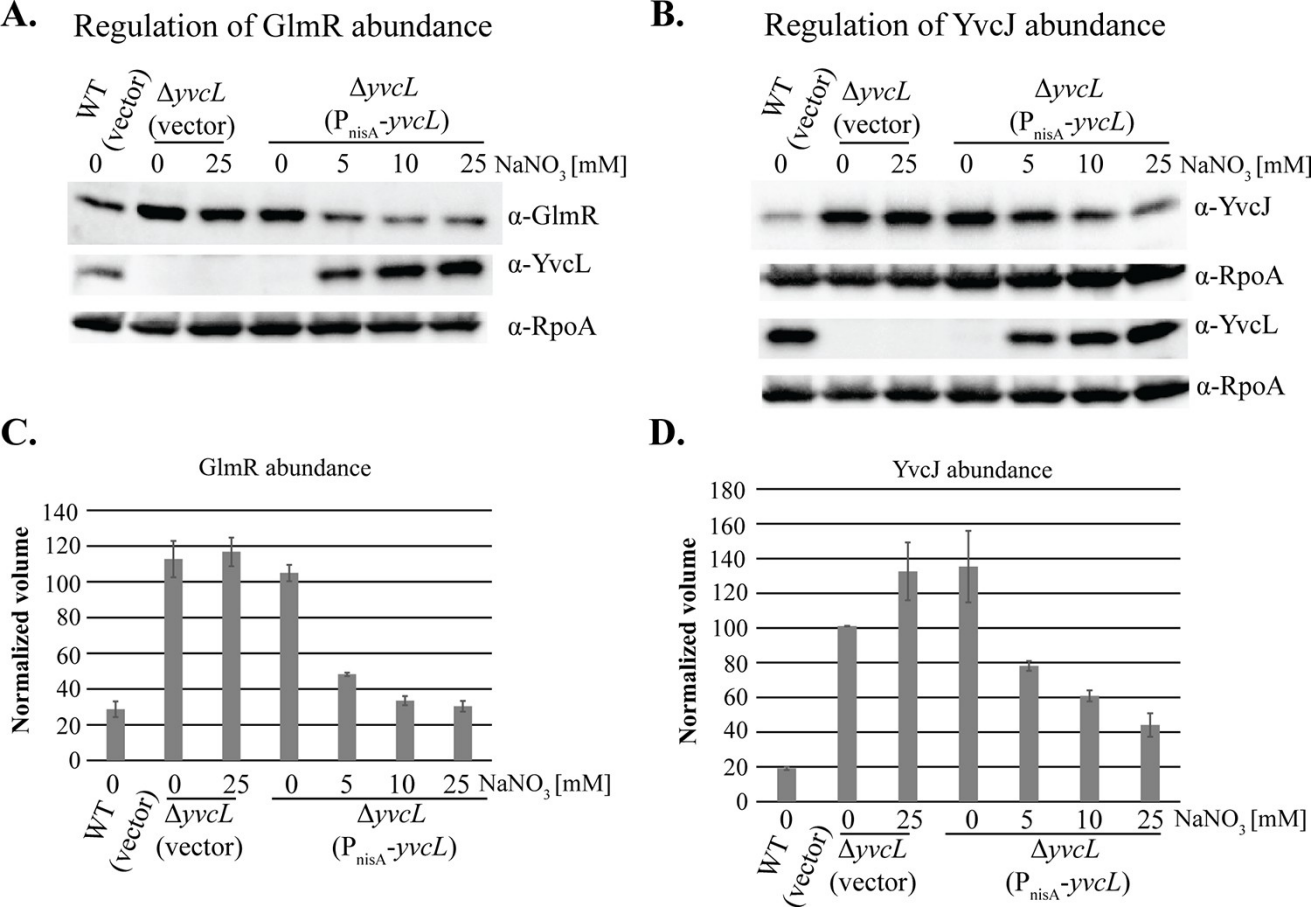
Abundance of GlmR and YvcJ is regulated by YvcL. Bacteria were grown to exponential phase in MH broth in presence or absence of different concentrations of NaNO_3_ inducer. Whole cell lysates were prepared, subjected to SDS-PAGE and abundance of GlmR (**A**) and YvcJ (**B**) assessed via immunoblot analysis. YvcJ and YvcL have the same molecular weights, so the same samples were analyzed separately to allow detection of both proteins. RpoA is a loading control. **(C, D)**. Quantification of GlmR abundance from panel A and YvcJ from panel B normalized to total protein in each lane from two biological replicates. Strains were: Wild-type (WT), *E*. *faecalis* OG1; Δ*yvcL*, DDJ260; Plasmids were: vector, pJLL286; P_nisA_-*yvcL*, pDDJ271.

*yvcL* encodes a putative DNA binding protein [35–37]. To understand what genes it might regulate, we constructed an in-frame *yvcL* deletion mutant of *E*. *faecalis* and subjected it to RNA-seq analysis. Twenty genes with altered expression (log_2_FC ≥ 1) when compared to otherwise isogenic wild-type *E*. *faecalis* OG1 were identified (S6 Table). Among them were *yvcJ* and *glmR*, which were both upregulated relative to wild-type. We validated the RNA-seq data for *yvcJ* and *glmR* using both RT-qPCR and immunoblotting, demonstrating elevated expression for both in the Δ*yvcL* mutant (S9 Fig). This phenotype could be complemented by inducible expression of YvcL from a plasmid in the Δ*yvcL* mutant (Fig 3), demonstrating that the Δ*yvcL* mutation is indeed responsible. Moreover, we found that expression of the *E*. *faecium* YvcL homolog in the *E*. *faecalis* OG1 Δ*yvcL* mutant demonstrated cross-complementation (S3 Table), indicating that the YvcL homologs from *E*. *faecalis* and *E*. *faecium* are functionally equivalent. Analysis of the Δ*yvcL* mutant for cephalosporin resistance revealed a 2-to-4-fold increase in resistance for second and third generation cephalosporins (Table 4), that could also be complemented by inducible expression of YvcL (S7 Table). The modest increase in resistance of the Δ*yvcL* mutant is consistent with the modest increase in GlmR levels, suggesting that GlmR was driving the elevated ceftriaxone resistance of the Δ*yvcL* mutant. To test this, we constructed a Δ*(glmR yvcL)* double mutant and found that ceftriaxone resistance of the double mutant was substantially reduced (Table 5), indicating that the effect of YvcL on cephalosporin resistance is indeed mediated by GlmR. As expected, expression of GlmR in the double mutant restored ceftriaxone resistance (S8 Table).

**Table 4:** Antimicrobial resistance of Δ*yvcJ*, Δ*glmR* and Δ*yvcL* mutants.

	MIC^a^ (μg/ml)			
	WT_OG1_	Δ*glmR*_OG1_	Δ*yvcJ*_OG1_	Δ*yvcL*_OG1_
*Cell wall targets*				
Ceftriaxone	64	8	512	128
Cefuroxime	64	8	512	256
Fosfomycin	32	32	32	32
Bacitracin	64	64	32	32
Vancomycin	2	2	2	2
D-cycloserine	128	128	128	128
Ampicillin	1	0.5	1	0.5
*Other targets*				
Chloramphenicol	8	8	8	8
Norfloxacin	4	4	2	4
Erythromycin	1	1	1	1

^a^Median minimal inhibitory concentrations for different antimicrobials was determined in MH broth after 24 h incubation at 37°C, from a minimum of three independent experiments.

Strains were: Wild-type (WT), *E*. *faecalis* OG1; Δ*glmR*, DDJ245; Δ*yvcJ*, DDJ326; Δ*yvcL*, DDJ260.

**Table 5:** Ceftriaxone resistance of single and double mutants.

Strain	MIC_ceftx_ (μg/ml)
**Wild-type**	64
**Δ*glmR***	8
**Δ*yvcJ***	512
**Δ*yvcL***	128
**Δ(*yvcJ-glmR)***	64
**Δ(*glmR-yvcL)***	4
***yvcJ***_**K18A**_	512

^a^Median minimal inhibitory concentrations for ceftriaxone (MIC_ceftx_) determined in MH broth after 24 h incubation at 37°C, from a minimum of three independent experiments. Strains were: Wild-type, *E*. *faecalis* OG1; Δ*glmR*, DDJ245; Δ*yvcL*, DDJ260; Δ*yvcJ*, DDJ326; Δ(*glmR*-*yvcL)*, DDJ332; Δ(*yvcJ-glmR)*, DDJ338; *yvcJ*_K18A_, DDJ446.

**Fig 4:**
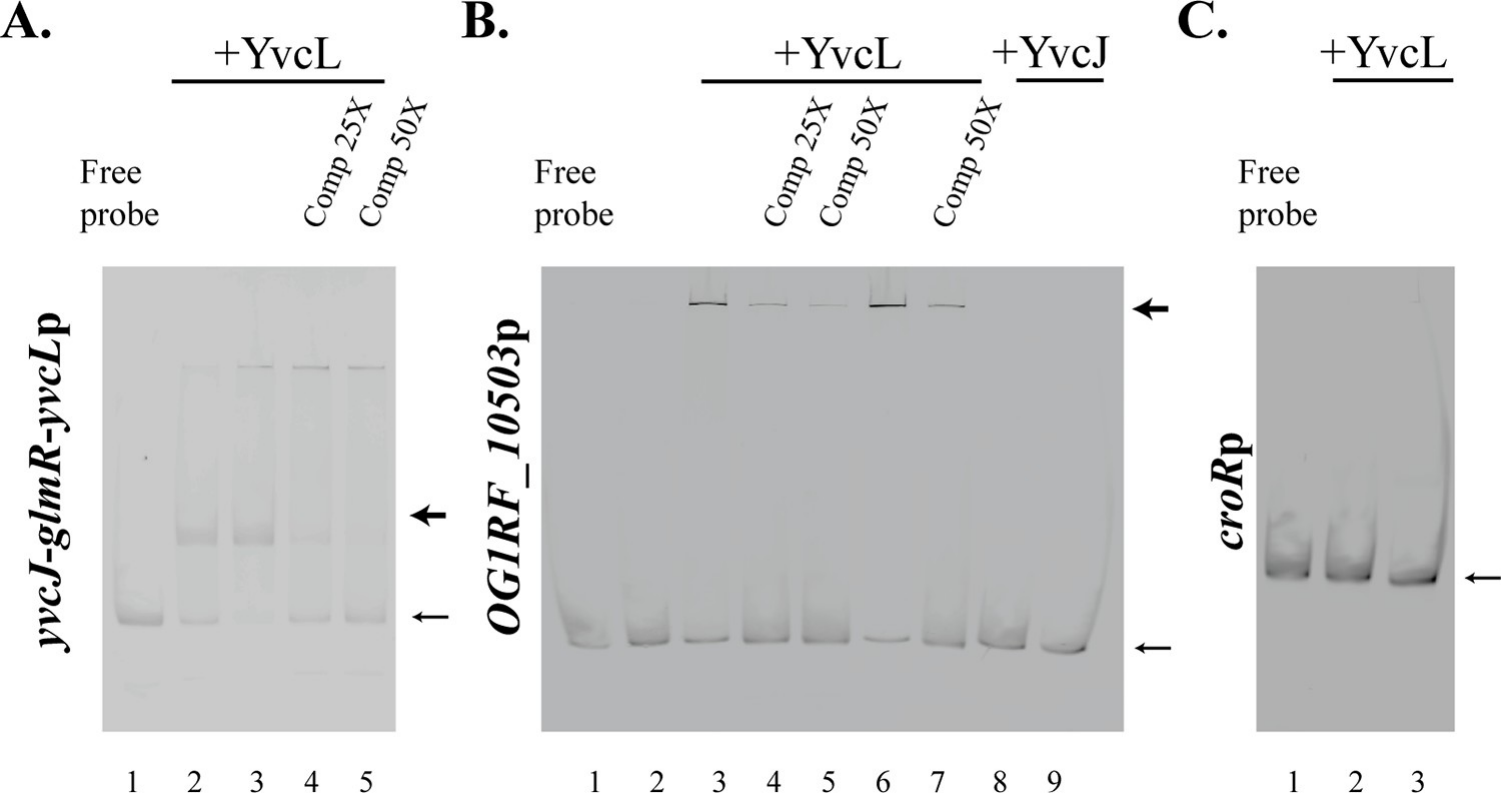
YvcL binds DNA. EMSAs were performed using Cy5-labeled probes containing putative promoter regions of *yvcJ*-*glmR*-*yvcL* (*yvcJ*-*glmR*-*yvcL*p) (A), *OG1RF_10503* (B), and a control gene (*croRp*) (C). Purified His_6_-SUMO-YvcL was incubated with probes and subjected to electrophoresis. *Comp*, unlabeled specific competitor for each probe at 25X or 50X excess as indicated. Thin arrows indicate the position of free probe, and thick arrows indicate the position of probe-DNA complexes. Representative results from at least three independent experiments are shown. Lanes 1: free probe; Lanes 2: 1μM YvcL; Lanes 3–5: 3 μM YvcL; Lanes 6–7: 5 μM YvcL; Lanes 8–9: 3 and 5 μM YvcJ, respectively.

Given that YvcL contains a putative DNA-binding domain, we hypothesized that this regulation might be mediated through YvcL binding to the intergenic region upstream of the *yvcJ-glmR-yvcL* operon. To address this possibility, we tested if recombinant, purified *E*. *faecalis* His_6_-SUMO-YvcL bound DNA *in vitro* using electrophoretic mobility shift assays (EMSAs). Cy5-labeled probes were prepared that encompassed three putative promoter regions: (i) that for the *yvcJ*-*glmR*-*yvcL* operon; (ii) that for *OG1RF_10503* (another gene with altered expression identified in the RNAseq analysis, S6 Table); or (iii) that for a control probe (the promoter region for the *croR* gene, which did not exhibit altered expression in the RNA-seq data). While no mobility shift was observed with the control probe, purified His_6_-SUMO-YvcL bound the putative promoter segment from the *yvcJ*-*glmR*-*yvcL* operon as indicated by the appearance of a shifted band in the EMSA (Fig 4). This interaction was specific because addition of excess unlabeled probe competed with the labeled probe for binding. For the putative promoter of *OG1RF_10503*, we observed signal in the wells of the gel when incubated with His_6_-SUMO-YvcL, suggesting a protein-DNA interaction for which the complex is too large to enter the gel (Fig 4). This interaction is also specific as addition of excess unlabeled probe eliminated this signal. Additionally, no such signal in the wells was observed when the probe was incubated with a control His_6_-SUMO-tagged protein with no known DNA binding activity (His_6_-SUMO-YvcJ), indicating that YvcL is likely regulating expression of *OG1RF_10503* by directly binding its promoter, consistent with the RNA-seq and RT-qPCR results. Taken together, the data suggest a model where YvcL negatively regulates expression of the *yvcJ*-*glmR*-*yvcL* operon and *OG1RF_10503* via direct binding to their promoter regions.

**Fig 5:**
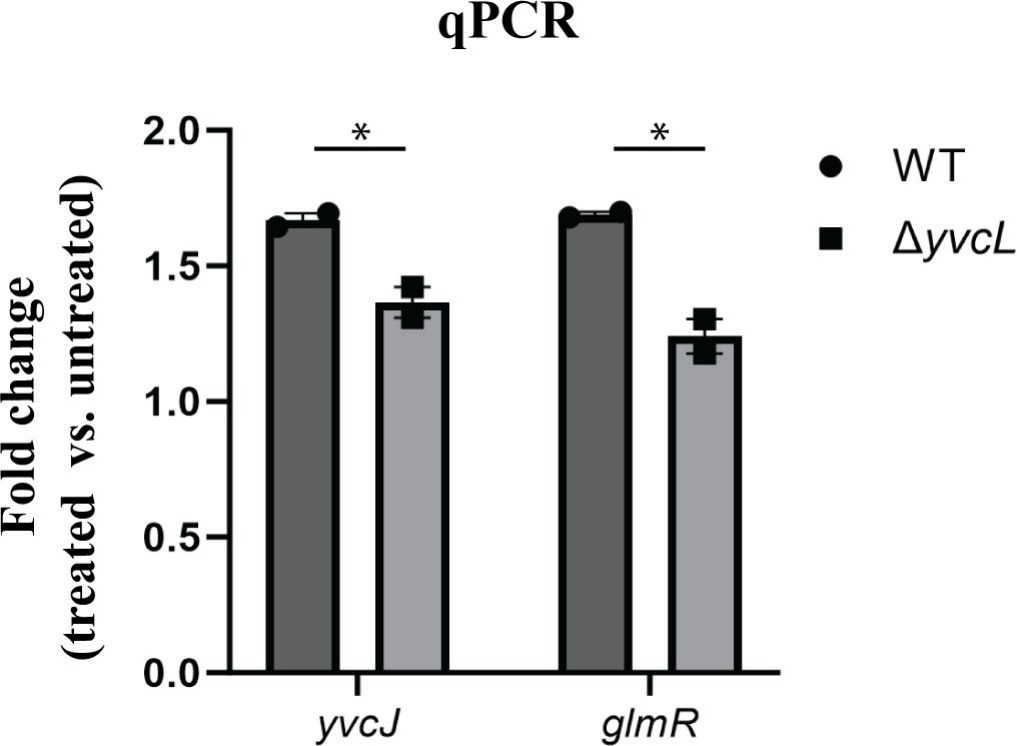
Vancomycin-induced increase in *yvcJ* and *glmR* expression is reduced in the Δ*yvcL* mutant. RNA was purified from *E*. *faecalis* strains grown to exponential phase in MH broth and treated, or not, with 3 μg/ml vancomycin. Expression was measured using RT-qPCR with normalization to 16S rRNA, and fold induction (treated versus untreated) was calculated. The data represent the mean ± standard error from two independent experiments, each performed in triplicate; *, p < 0.05 as determined using a *t* test. Strains were: Wild-type, *E*. *faecalis* OG1; Δ*yvcL*, DDJ260.

Given that GlmR impacts enterococcal cephalosporin resistance, we hypothesized that expression of the *yvcJ*-*glmR*-*yvcL* operon might be induced in wild-type cells upon exposure to cell wall stress. To test this, we exposed exponentially growing *E*. *faecalis* cells to vancomycin (a cell-wall-targeting antibiotic that is known to be a robust inducer of enterococcal signaling systems that respond to cell wall stress; [38–40]) and monitored *yvcJ* and *glmR* expression via RT-qPCR. We observed a modest increase in expression for both genes upon vancomycin exposure of wild-type cells (Fig 5) that was similar in magnitude to the increase observed upon deletion of *yvcL*. The magnitude of the vancomycin-induced increase in expression of both genes was reduced in the Δ*yvcL* mutant, suggesting that in wild-type cells YvcL responds to a signal associated with cell wall stress, resulting in de-repression of the *yvcJ*-*glmR*-*yvcL* operon.

### YvcJ and GlmR reciprocally regulate cephalosporin resistance

Given the impact of GlmR on cephalosporin resistance, and the conservation of the *yvcJ-glmR-yvcL* gene cluster, we reasoned that YvcJ might also impact cephalosporin resistance. To test this, we constructed a mutant lacking *yvcJ* in *E*. *faecalis*. Because of the overlap between the *yvcJ* and *glmR* genes and therefore the possibility that they are translationally coupled, we retained the last 50 codons of *yvcJ* in our in-frame deletion construct for *yvcJ*, to avoid perturbing expression of *glmR* (which we verified by immunoblot; S9 Fig). The resulting Δ*yvcJ* mutant exhibited a hyper-resistant phenotype specifically towards second and third generation cephalosporins (Table 4), which could be complemented with inducible expression of YvcJ (S10 Fig) from a plasmid (S7 Table). Given that GlmR expression is unchanged in the Δ*yvcJ* mutant (S9 Fig), these results indicate that YvcJ impacts cephalosporin resistance in a reciprocal manner compared to GlmR, namely by negatively regulating phenotypic resistance. Moreover, we found that expression of the *E*. *faecium* YvcJ homolog in the *E*. *faecalis* OG1 Δ*yvcJ* mutant demonstrated cross-complementation (S3 Table), indicating that the YvcJ homologs from *E*. *faecalis* and *E*. *faecium* are functionally equivalent.

To determine if YvcJ exerts its impact on cephalosporin resistance by inhibiting GlmR activity (or conversely, if GlmR acts on YvcJ), we performed a test for epistasis by simultaneously deleting both genes. The resulting Δ(*yvcJ glmR)* double mutant exhibited a ceftriaxone resistance phenotype that was distinct from either of the single mutants; namely, the level of ceftriaxone resistance for the double mutant was intermediate relative to either of the single mutants (Table 5). Because the phenotype of the double mutant differed from either of the single mutants, these results indicate that YvcJ and GlmR do not act only on each other to impact resistance, but rather antagonize each other’s activity, at least in part, through a different mechanism. Recently we reported that increased abundance of the peptidoglycan synthesis protein MurAA can drive elevated cephalosporin resistance [20,41], but immunoblotting revealed no difference in MurAA abundance in the double mutant (S11 Fig).

### Regulation by YvcJ and GlmR promotes fitness during cephalosporin stress

**Fig 6:**
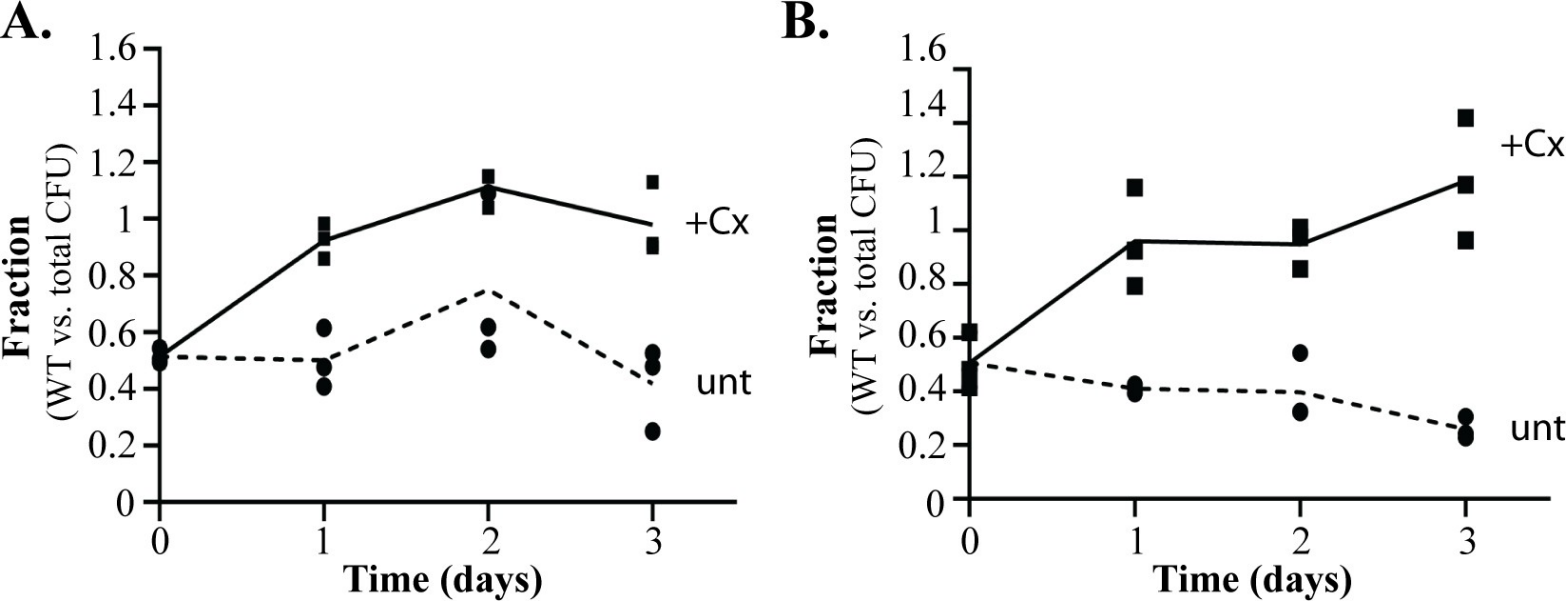
The Δ(*yvcJ glmR*) double mutant exhibits a fitness defect in co-culture with wild-type when exposed to ceftriaxone. *E*. *faecalis* strains were mixed in equal proportions and cocultured in MH broth without (dashed line; unt, untreated) or with 4 μg/ml ceftriaxone (full line; Cx, ceftriaxone) with daily repeated cycles of growth and dilution. Samples were plated for CFU at Day 0, 1, 2 and 3 on MH agar plates supplemented, or not, with spectinomycin (A) or fusidic acid (B) and fraction of marked (wild-type) strain versus the total CFU was calculated. Data represents three biological replicates. Strains in A were: OG1Sp, spectinomycin resistant wild-type (WT); DDJ338, unmarked Δ(*yvcJ glmR*). Strains in B were CK138, fusidic acid resistant wild-type; DDJ338, unmarked Δ(*yvcJ glmR*).

Although YvcJ and GlmR individually impact enterococcal cephalosporin resistance in a reciprocal manner, the ceftriaxone MIC of the Δ(*yvcJ glmR)* double mutant mimics that of otherwise wild-type cells (Table 5). Because the *yvcJ-glmR-yvcL* operon has been conserved among Gram-positive bacteria through evolution, we reasoned that YvcJ/GlmR-dependent regulation must provide a fitness benefit to *E*. *faecalis* cells under some circumstances. To test for a fitness effect, we performed co-culture competition experiments in which the Δ(*yvcJ glmR)* double mutant was co-cultured with wild-type cells in the presence or absence of a sub-inhibitory concentration of ceftriaxone (4 μg/ml; 1/16^th^ MIC for both strains). The wild-type cells were marked with an unrelated antibiotic resistance marker to distinguish them from the otherwise unmarked deletion mutant cells. Additionally, two such marked wild-type strains with different antibiotic resistance markers (fusidic acid or spectinomycin) were used in different experiments to ensure that the resistance marker allele in wild-type cells did not impact the outcome. Regardless of the resistance marker used to identify wild-type cells, in both cases co-culture of the mutant and wild-type over 3 days in the absence of ceftriaxone did not result in a substantial change in the relative proportions of each strain (Fig 6). However, in the presence of subinhibitory ceftriaxone, the wild-type OG1 cells rapidly outcompeted the Δ(*yvcJ glmR)* double mutant such that almost all of the cells were wild-type after only 1 day of co-culture. This outcome was the case regardless of the resistance marker used to identify the wild-type cells, indicating that reciprocal regulation by YvcJ/GlmR provides a fitness benefit to *E*. *faecalis* during cephalosporin stress.

### UDP-GlcNAc binding is critical for YvcJ function *in vivo*

**Fig 7:**
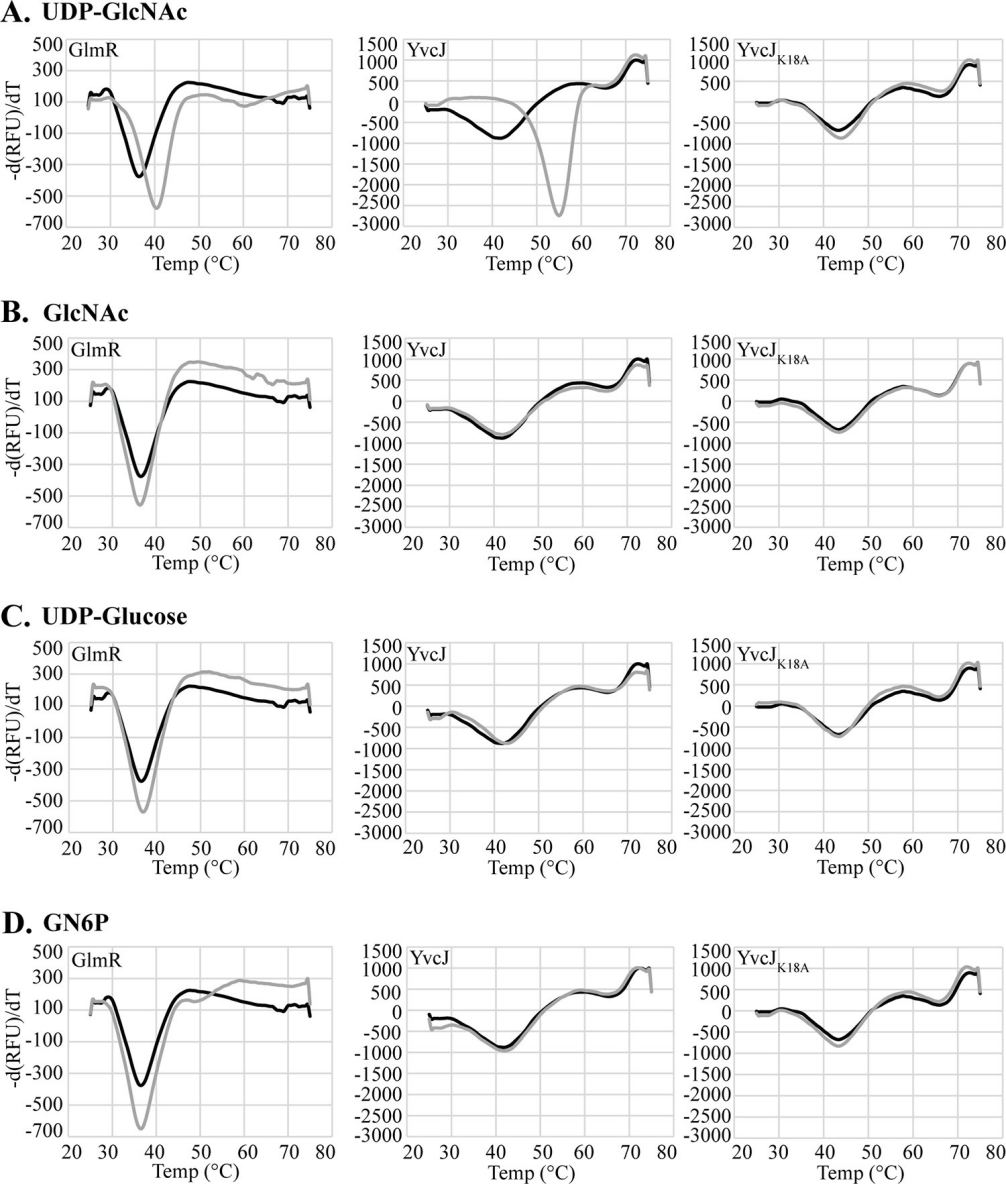
GlmR and YvcJ bind UDP-GlcNAc. Thermal shift assay (TSA) was performed using 10 μM purified protein in the absence of compound (black line) or presence of 1mM compound of interest (gray line). Melting temperature profiles for each protein/compound combination were determined by plotting the first derivative of the fluorescence values. UDP-GlcNAc, uridine diphosphate N-acetylglucosamine (A); GlcNAc, N-acetylglucosamine (B); UDP-Glucose, uridine diphosphate glucose (C); GN6P, glucosamine 6-phosphate (D).

YvcJ and GlmR of *B*. *subtilis* have been shown to bind to UDP-GlcNAc [21,31], suggesting a potential mechanism to regulate their activity in response to changes in the metabolic state of the cell. To determine if enterococcal YvcJ and GlmR also could bind UDP-GlcNAc, we expressed and purified recombinant forms of each and used thermal shift assays (TSAs) to test for binding *in vitro*, as previously described [21]. Thermal denaturation of purified recombinant YvcJ or GlmR was monitored in the presence or absence of candidate ligands using SYPRO Orange dye. Changes in the thermal denaturation temperature of a protein can occur upon binding of a ligand, reflecting differences in stability in the bound vs. unbound state (*i*.*e*. the thermal shift). When incubated with UDP-GlcNAc, both YvcJ and GlmR exhibited clear thermal shifts that did not occur when incubated with UDP-glucose, GlcNAc, or glucosamine-6-P (Fig 7), confirming that both YvcJ and GlmR from *E*. *faecalis* can bind UDP-GlcNAc. Additionally, we found that both the GlmR D42A D43A and GlmR N206A mutants, predicted to be impaired at uridyltransferase activity, retained the ability to bind UDP-GlcNAc (S7B Fig).

To determine if UDP-GlcNAc binding is functionally important for YvcJ/GlmR-mediated regulation of cephalosporin resistance, we sought to identify YvcJ or GlmR variants that were unable to bind UDP-GlcNAc. Specific amino acid residues important for binding of *B*. *subtilis* GlmR to UDP-GlcNAc have been described, of which Y265 and R301 are the most critical [21]. However, neither of those residues are conserved in the primary amino acid sequence of *E*. *faecalis* GlmR (S12 Fig). Thus, more work will be required to identify specific residues of *E*. *faecalis* GlmR that contribute to UDP-GlcNAc binding. In contrast, a residue required for UDP-GlcNAc binding by YvcJ of *B*. *subtilis* (K22; [31]) is conserved in the *E*. *faecalis* YvcJ sequence (K18 in *E*. *faecalis* YvcJ). To test for a role of YvcJ K18 in UDP-GlcNAc binding, we expressed and purified a recombinant K18A variant of *E*. *faecalis* YvcJ. In TSAs, the K18A variant exhibited a melting temperature that was identical to that of wild-type YvcJ, indicating that the K18A mutation did not inherently destabilize the protein. However, unlike wild-type YvcJ, no shift was observed upon incubation of the YvcJ K18A variant with UDP-GlcNAc (Fig 7), indicating a loss of UDP-GlcNAc binding ability for the K18A mutant.

To determine if UDP-GlcNAc binding is important for YvcJ function *in vivo*, we introduced the K18A substitution into the chromosomal copy of *yvcJ* in *E*. *faecalis* OG1, thereby maintaining normal control of expression from the *yvcJ-glmR-yvcL* operon. Immunoblotting confirmed that both YvcJ K18A and GlmR were expressed at wild-type levels in the resulting mutant (S13 Fig). However, antimicrobial susceptibility assays revealed that the YvcJ K18A mutant was hyper-resistant to ceftriaxone, with an MIC value equivalent to the *yvcJ* deletion mutant (Table 5). These results indicate that YvcJ K18A could no longer negatively regulate ceftriaxone resistance, and suggest that UDP-GlcNAc binding is essential for the ability of YvcJ to do so.

## Discussion

GlmR has been identified as an important regulator of cell wall homeostasis and resistance to cell-wall-active antibiotics in several Gram-positive bacteria [21–27]. In *B*. *subtilis*, GlmR acts to boost the activity of GlmS thereby enhancing diversion of metabolites into the cell wall biosynthesis pathway [27], likely explaining the role of GlmR in cell wall homeostasis as well as its requirement for growth of *B*. *subtilis* on gluconeogenic carbon sources. GlmR from several Gram-positive bacteria catalyzes conversion of GlcNAc-1P + UTP to UDP-GlcNAc [28], again implicating GlmR in biosynthesis of the cell wall. The biological roles of the genes flanking *glmR* in the chromosome, *yvcJ* and *yvcL*, are less clear, although YvcJ of *B*. *subtilis* has been proposed to regulate GlmR function through direct protein-protein interactions [31]. The functions of YvcJ, GlmR, and YvcL in enterococci are largely unknown. Our results support a model in which expression of the *yvcJ-glmR-yvcL* operon is regulated in response to cell wall stress by YvcL. GlmR and YvcJ antagonistically regulate intrinsic resistance to cephalosporins, which provides a fitness benefit to *E*. *faecalis* during exposure to cephalosporins. This regulation appears to be responsive to the cell-wall-synthesis pathway metabolite UDP-GlcNAc, possibly as a means of sensing the status of cell wall synthesis. Moreover, our data indicate that GlmR of *E*. *faecalis* likely regulates the response to cell wall stress through mechanisms that are distinct from the well-studied GlmR of *B*. *subtilis*, suggesting that the GlmR-YvcJ regulatory module may have evolved unique targets commensurate with the particular needs of the specific host bacterial species in which it resides.

The YvcL protein contains a putative DNA-binding domain, suggesting a main function as a transcriptional regulator. Our RNA-seq analysis of the *yvcL* deletion mutant revealed a small set of 20 differentially regulated genes, among which we found *yvcJ* and *glmR* to be upregulated upon loss of *yvcL* (S6 Table). The RNA-seq data was validated by both RT-qPCR and immunoblotting (S6 Table AND S9 Fig), confirming that YvcL negatively regulates expression of the *yvcJ-glmR-yvcL* operon, presumably through direct binding to the promoter (Fig 4). Additionally, we found that enhanced expression of the *yvcJ-glmR-yvcL* operon occurred upon exposure to antibiotic-mediated cell wall stress (vancomycin), and the vancomycin-induced increase was diminished in the *yvcL* mutant (Fig 5), implicating YvcL in transcriptional control of the *yvcJ-glmR-yvcL* operon in response to cell wall stress. The nature of the molecular signal that modulates YvcL repression of the operon during cell wall stress remains unknown and will be a focus for future research.

The *yvcJ* and *glmR* genes overlap by a nucleotide, implying that YvcJ and GlmR might be functionally related. We found that nonpolar deletions of *glmR* or *yvcJ* both impact cephalosporin resistance, but in opposite directions–deletion of *glmR* leads to a loss of ceftriaxone resistance, while deletion of *yvcJ* leads to elevated ceftriaxone resistance (Table 5). It does not appear that YvcJ or GlmR exerts its effect on ceftriaxone resistance exclusively by modulating the activity of the other, because a double mutant lacking both *yvcJ* and *glmR* exhibits an intermediate level of ceftriaxone resistance that is different from either of the single mutants (Table 5). Thus, although YvcJ and GlmR antagonistically influence ceftriaxone resistance, they must do so–at least in part–through their action on some other as-yet-unidentified determinant of cephalosporin resistance. Studies of *B*. *subtilis* GlmR indicate that it can boost the activity of GlmS to enhance the flow of metabolites into the cell wall biosynthesis pathway, because overexpression of GlmS or provision of GlcNAc to cells (which can be imported and converted to the GlmS reaction product) can rescue phenotypic defects of a *B*. *subtilis glmR* mutant [27]. Our results suggest that GlmR (and presumably YvcJ) of *E*. *faecalis* do not act by this mechanism to regulate ceftriaxone resistance or cell wall homeostasis, because: (1) overexpression of GlmS does not rescue the ceftriaxone resistance defect of the *E*. *faecalis glmR* mutant (S5 Table); (2) provision of GlcNAc does not rescue the ceftriaxone resistance defect of the *glmR* mutant (S4 Table), despite the fact that exogenous GlcNAc can be used as a substrate for growth (S5 Fig); and (3) the enterococcal *glmR* mutants do not exhibit a growth defect in gluconeogenic medium like *glmR* mutants of *B*. *subtilis* (S5 Fig). Collectively these results suggest the GlmR/YvcJ regulatory module has evolved to regulate distinct targets in enterococci relative to *B*. *subtilis*.

While the specific molecular target(s) for regulation by GlmR/YvcJ remain unknown, the fine-tuned control of cephalosporin resistance they provide is essential for competitive fitness during exposure to cephalosporins. Co-culture of wild-type with the *yvcJ*-*glmR* double deletion mutant–which exhibit identical MIC values for ceftriaxone–revealed that wild-type and the mutant were similarly competitive in the absence of ceftriaxone stress over 3 days (Fig 6), consistent with the lack of any growth defect for the mutant in MHB. However, in the presence of a low, sub-inhibitory concentration of ceftriaxone (1/16^th^ the MIC), the mutant was rapidly outcompeted by wild-type (Fig 6). This was true for each of 2 different wild-type strains that were tested (each marked with a different antibiotic resistance marker, to ensure that the resistance marker did not have an effect). Thus, despite the fact that the *yvcJ*-*glmR* double mutant exhibits a similar MIC to wild-type when analyzed in monoculture, proper regulation of cephalosporin resistance by GlmR/YvcJ is nevertheless essential in competitive environments and provides an evolutionary rationale for the conservation of the *yvcJ-glmR-yvcL* gene cluster among enterococcal genomes.

YvcJ and GlmR from *B*. *subtilis* have previously been shown to bind the cell-wall-biosynthesis pathway metabolite UDP-GlcNAc [21,31], which was shown to be essential for GlmR-YvcJ interaction. Using recombinant purified proteins, we confirmed that both YvcJ and GlmR from *E*. *faecalis* could also bind UDP-GlcNAc (Fig 7). Binding of UDP-GlcNAc appeared relatively specific, as substantial shifts were not observed for related ligands, but formally our data do not exclude the possibility that GlmR and YvcJ might also bind these other ligands with lower affinity than UDP-GlcNAc. To test for physiological relevance of UDP-GlcNAc binding *in vivo*, we constructed a variant of YvcJ that was stably folded but no longer able to bind UDP-GlcNAc (K18A). Despite being expressed at wild-type levels in *E*. *faecalis* cells, YvcJ K18A was nonfunctional (Table 5), suggesting that UDP-GlcNAc binding is critical for regulation of YvcJ activity. Previously we showed that flow of metabolites through the PG biosynthesis pathway is an important determinant of cephalosporin resistance in *E*. *faecalis* [20]. Binding of UDP-GlcNAc by YvcJ to modulate its activity might therefore be one mechanism by which the cell can monitor the status of cell wall synthesis to regulate cephalosporin resistance and potentially peptidoglycan synthesis more generally. Future studies to examine the ultrastructure of the cell wall as a function of YvcJ / GlmR deletion or overexpression may help shed light on their coordinated impact on the enterococcal cell wall.

## Methods

### Bacterial strains and growth conditions

The bacterial strains and plasmids used in this study are listed in S9 Table. Oligonucleotides were synthesized by Integrated DNA Technologies, Inc. *E*. *coli* strains were grown in either Lysogeny broth (LB) or half-strength brain heart infusion (hBHI) (BD), at 30°C or 37°C, as appropriate, with shaking at 225 rpm. *E*. *faecalis* was cultured in Mueller-Hinton broth (MHB) and grown at 30°C unless otherwise specified, with shaking at 225 rpm. The solid medium used is the same as above with addition of 1.6% (wt/vol) Bacto agar (Difco). When required, antibiotics were added at the following final concentrations: for *E*. *coli*, erythromycin (Em), 100 μg/ml; chloramphenicol (Cm), 10 μg/ml; for *E*. *faecalis*, both erythromycin and chloramphenicol were used at 10 μg/ml.

### Genetic manipulation of enterococci and plasmid construction

Gene deletion or introduction of mutant alleles on the chromosome was performed using the temperature-sensitive, counter-selectable allelic exchange plasmids pCJK245 [42] or pJH086 [40]. Upstream and downstream regions of the gene of interest were amplified from genomic DNA by PCR and introduced into appropriate plasmids using Gibson assembly [43]. Deletion alleles retain a variable number of codons at the beginning and end of each gene to avoid polar effects on adjacent genes and specific details of each construct are listed in S9 Table. All mutants were constructed independently, at least twice and analyzed to ensure agreement in observed phenotypes. For plasmid construction, genes of interest were amplified by PCR and introduced into appropriate plasmids using Gibson assembly [43], incorporating a synthetic ribosome-binding site (AGGAGGAACTCAAT) upstream of the gene open reading frame when necessary. Locus tags: *yvcJ*_Efs_, OG1RF_10500; *glmR*_Efs_, OG1RF_10501 (EF0767 in V583); *yvcL*_Efs_, OG1RF_10502; *yvcJ*_Efm_, EFSG_RS09470; *glmR*_Efm_, EFSG_RS09475; *yvcL*_Efm_, EFSG_RS09480.

### Antimicrobial susceptibility and growth assays

Minimal inhibitory concentrations (MICs) were determined using a broth microdilution method with 2-fold serial dilutions of antibiotics prior to inoculation of normalized bacterial samples from stationary-phase cultures. Cultures were normalized to optical density at 600 nm (OD_600_) of 4 x 10^−5^ (∼1 × 10^5^ CFU for the wild type). Assays were carried out in MHB (supplemented with Cm or Em, as necessary, for maintenance of plasmids; nitrate (NaNO_3_) was supplemented when inducible vectors were used). Plates were incubated in a Bioscreen C plate reader at 37°C for 24 h with brief shaking before each measurement. The OD_600_ was measured every 15 min, and the lowest concentration of antibiotic that prevented growth was recorded as the MIC. Growth curves were obtained from samples without antibiotic present.

### Immunoblot analysis

Stationary-phase cultures of strains grown at 30°C, 225 rpm were diluted to an OD_600_ = 0.01 in MHB (supplemented with Cm or Em as appropriate; nitrate was used when inducible vectors were present at concentrations indicated in figures) and incubated at 37°C with shaking until the exponential phase (OD_600_ = ∼0.2). Cultures were mixed with an equal volume of EtOH/acetone (1:1), collected by centrifugation, and washed once with water. To prepare cell lysates, bacteria were suspended in lysozyme buffer (10 mM Tris [pH 8], 50 mM NaCl, 20% sucrose), normalized, treated with lysozyme (5 mg/ml) for 30 min at 37°C, and mixed with Laemmli SDS sample buffer supplemented with dithiothreitol (DTT, GoldBio), boiled 5 min. Samples were then subjected to SDS-PAGE, where gels were supplemented with 2,2 trichloroethanol (TCE, Sigma) to allow for total protein visualization using a ChemiDoc (BioRad). After SDS-PAGE, proteins were transferred to nitrocellulose membrane (Bio-Rad) and probed with rabbit anti-GlmR, rabbit anti-YvcJ, rabbit anti-YvcL, rabbit anti-MurAA, and rabbit anti-RpoA antisera (custom polyclonal antisera). Detection was performed with goat anti-rabbit horseradish peroxidase (HRP)-conjugated secondary antibody (Invitrogen).

### RNAseq sample preparation and analysis

Quadruplicate cultures of wild-type OG1 and Δ*yvcL* mutant were grown in MHB, 37°C, 225 rpm until exponential phase (OD_600_ = ~0.2). Cultures were mixed with equal volume of cold ethyl alcohol (EtOH)-acetone (1:1) to kill the bacteria and preserve the RNA and harvested by centrifugation, 10 min, 4,500 rpm, 4°C. Pellets were subsequently resuspended in water to wash and transfer to 1.5 ml tubes and samples centrifuged again 1 min, 15,000 rpm prior to removal of supernatant. RNA was purified using Total RNA Mini Kit (Blood/Cultured Cell) (IBI Scientific). Turbo DNase (Invitrogen) was used to remove any carryover DNA prior to phenol/chloroform extraction (using phenol:chloroform:isoamyl alcohol mix from Ambion and Phase Lock Gel Heavy tubes from 5PRIME) and ethanol precipitation of the resulting RNA. Depletion of rRNA was done using RiboMinus Bacteria 2.0 Transcriptome Isolation Kit (ThermoFisher) prior to library preparation and sequencing at the University of Minnesota Genomics Center. Bioinformatic analysis of RNAseq data: raw reads were checked for quality using FastQC v. 0.11.9 (https://github.com/s-andrews/FastQC) and trimmed using TrimGalore! v.0.6.6 using default parameters (https://github.com/FelixKrueger/TrimGalore). Bowtie2 v. 2.3.4.3 was used to build a reference to the RefSeq OG1RF reference genome (Accession NC_017316.1)[44]. Trimmed reads were then mapped back to the reference using Bowetie2, converted to BAM files with Samtools v. 1.11, and resulting BAM files were sorted [45,46]. The featureCounts tool was then used to count the number of reads per gene using these flags: -p -s 2, -t gene, -g gene_id, and -a [47]. The resulting counts files were then imported into R to run through the DESeq2 pipeline to determine differentially expressed genes [48–50].

### Operon and gene expression analysis

Stationary-phase cultures of *E*. *faecalis* strains were diluted to an OD_600_ = 0.01 in MHB. Cultures were grown at 37°C to exponential phase (OD_600_ = ∼0.2), samples collected by mixing with equal volume of cold EtOH/Acetone (1:1) to kill the bacteria and preserve the RNA and harvested by centrifugation. RNA isolation was done according to manufacturer directions, using Total RNA Mini Kit (Blood/ Cultured Cell) (IBI Scientific). RT-PCR analysis was performed on RNA isolated from wild-type *E*. *faecalis* using primers that spanned *OG1RF_10499*-*yvcJ*, *yvcJ*-*glmR* and *glmR*-*yvcL*, *yvcL*-*OG1RF_10503* regions generating ~350 bp amplicons if transcripts were present; genomic OG1RF DNA was used as a control. Quantitative reverse transcription-PCR (RT-qPCR) was performed on RNA extracted from at least two biological replicates of each sample and assessed in technical triplicates. Calculations of the fold change in gene expression used the Pfaffl method and 16S rRNA as a reference gene.

### Competition assay

Stationary-phase cultures of *E*. *faecalis* strains were normalized and mixed 1:1 in the following manner: a) OG1Sp:DDJ338, b) CK138:DDJ338 using two independent biological replicates of each culture. Ten-fold serial dilutions of the mixed cultures were then prepared in a 96-well plate and plated for CFU enumeration on: a) MHB and MHB supplemented with spectinomycin 500 μg/ml for OG1SP containing co-culture, or b) MHB and MHB supplemented with 25 μg/ml fusidic acid for the CK138 containing co-culture. The co-cultures were diluted to 1x10^-4^ starting inocula in MHB and MHB supplemented with 4 μg/ml ceftriaxone and incubated at 37°C, overnight, 225 rpm. The mixed cultures were diluted and incubated each day and enumerated as above. Ratio of wild-type CFU (from MHB spectinomycin or fusidic acid agar plates) to total CFU (from MHB agar plates) was determined to assess fitness of the mutant relative to the wild-type.

### Overexpression and purification of *E*. *faecalis* YvcJ, GlmR and YvcL

Overnight cultures of *E*. *coli* BL21 [DE3] (pDDJ291, for YvcJ or pDDJ331 for YvcJ_K18A_), BL21 [DE3] (pDDJ237, for GlmR; pDDJ311, for GlmR_N206A_; or pDDJ313, for GlmR_D42-43A_), BL21 [DE3] (pDDJ292, for YvcL) were grown in LB supplemented with 50 μg/ml kanamycin at 37°C, diluted 50-fold into 200 ml of the same medium, incubated 3 h at 37°C at 225 rpm and then induced with 1 mM IPTG (isopropyl-β-D-thiogalactopyranoside) (Gold Biotechnology) for 3 h at 37°C for YvcJ and GlmR and 0.5 mM IPTG, 16°C, 225 rpm overnight for YvcL. Bacteria were collected by centrifugation (10,000 rpm, 10 min, 4°C) and cell pellets resuspended in 10 ml binding buffer (50 mM Tris, 300 mM NaCl, 5 mM imidazole, pH 8). Cell were disrupted by passing through French press 4X, and debris was removed by centrifugation (15,000 rpm, 20 min, 4°C) followed by filtration of supernatant through a 0.22-μm-pore-size filter. The filtered supernatant was loaded onto an Ni column (Profinity IMAC Ni-charged resin; Bio-Rad) equilibrated with binding buffer. Columns were subsequently washed with 5 column volumes of wash buffer (50 mM Tris, 300 mM NaCl, 20 mM imidazole, pH 8) and eluted in elution buffer (50 mM Tris, 300 mM NaCl, 500 mM imidazole, pH 8). Eluted fractions were analyzed by 10% SDS-PAGE, and fractions containing the protein were dialyzed against the following dialysis buffers: 1) 50 mM Tris pH8, 150 mM NaCl, 5% glycerol, for GlmR and corresponding mutants, 2) 100 mM MOPS [pH 7.5], 0.8 M NaCl, 1 mM EDTA, 5% glycerol for YvcJ and YvcL used in EMSA, 3) 50 mM Tris pH8, 150 mM NaCl, 5mM MgCl_2_, 1 mM EDTA, 2 mM DTT, 5% glycerol, for YvcJ used in thermal shift assay. Dialyzed proteins were stored in aliquots at −80°C.

## Electrophoretic mobility shift assay (EMSA)

Putative promoter segments of *yvcJ*-*glmR*-*yvcL* operon (*yvcJ*-*glmR*-*yvvL*p), *OG1RF_10503* (*OG1RF_10503*p), were amplified from *E*. *faecalis* OG1RF genomic DNA using an unlabeled forward primer and a Cy5-labeled reverse primer (IDT). These segments included the upstream region as well as a few nucleotides into the actual reading frame giving rise to ~275 bp fragments; in the case of the *croR* control probe (*croRp*), a known regulatory promoter region was amplified. Cy5-labeled probes (6.6 nM) and purified His_6_-SUMO-YvcL (1, 3 or 5 μM) or His_6_-SUMO-YvcJ (3 or 5 μM) were incubated in binding buffer (0.1 M MOPS [pH 7.5], 0.8 M NaCl, 1 mM EDTA, 1 mM MgCl_2_, 5% glycerol) supplemented with 1 μg poly(dA-dT), for 15 min at room temperature (RT). Loading dye was added and samples were subjected to electrophoresis at 80 V for 2.5 h at 4°C on a 6% polyacrylamide gel in 0.5 × Tris-borate-EDTA that had been pre-run for 15 min at 120 V. After electrophoresis, gels were imaged using the Typhoon imager (Amersham).

### Thermal shift assay

Thermal shift assay (TSA) was performed essentially as described previously [21]. In brief, purified recombinant proteins (10 μM) were mixed with SYPRO Orange (Invitrogen) in the presence or absence of compounds (1 mM) as indicated in the figure legend. Mixtures were heated at a rate 0.5°C/10 sec to 75°C using a Bio-Rad CFX Connect Real-Time PCR instrument and fluorescence measurements were taken at each 0.5°C increment. The first derivative of the fluorescence emission was plotted as a function of temperature and the T_m_ was determined as the lowest point of the curve. All thermal shift measurements were conducted in triplicate.

## Supporting information

S1 Table Supplementation with magnesium chloride does not enhance ceftriaxone resistance of the Δ*glmR* mutant. (DOCX)

S2 Table Complementation with *E*. *faecalis* GlmR from a plasmid. (DOCX)

S3 Table Cross-complementation of cephalosporin resistance of *E*. *faecalis* mutants using *E*. *faecium* genes. (DOCX)

S4 Table Supplementation with N-acetylglucosamine does not enhance ceftriaxone resistance. (DOCX)

S5 Table Overexpression of GlmS, GlmM, or GlmU does not enhance ceftriaxone resistance of Δ*glmR* mutant. (DOCX)

S6 Table Differentially expressed genes identified from RNASeq analysis of the *yvcL* deletion mutant reveals regulation of *yvcJ* and *glmR*. (DOCX)

S7 Table Complementation of Δ*yvcJ* and Δ*yvcL* mutants rescues ceftriaxone resistance to wild-type level. (DOCX)

S8 Table Ectopic expression of GlmR restores resistance of the Δ(*glmR yvcL*) mutant. (DOCX)

S9 Table Strains and plasmids used in this study. (DOCX)

S1 Fig GlmR is overexpressed from a plasmid. Expression of GlmR from the *E*. *faecalis* chromosome was compared to expression from a plasmid in both wild-type (WT) and Δ*glmR* mutant. Whole-cell lysates from *E*. *faecalis* cells grown exponentially in MH broth (+/- chloramphenicol for maintenance of plasmids) were subjected to immunoblot analysis for GlmR or RpoA (loading control). Strains and plasmids used were: WT, OG1; Δ*glmR*, DDJ245; vector, pJRG9; P-*glmR*, pJLL238.
(PDF)

S2 Fig Dose-dependent increase in GlmR abundance upon induction with nitrate. Whole-cell lysates from *E*. *faecalis* cells grown exponentially in MH broth (+/- erythromycin for maintenance of plasmids and listed nitrate concentrations) were subjected to immunoblot analysis for GlmR or RpoA (loading control). Strains and plasmids used were: wild-type (WT), OG1; Δ*glmR*, DDJ245; vector, pJLL286; P_nisA_-*glmR*, pDDJ262.
(PDF)

S3 Fig Lower expression of GlmR is observed from nitrate-inducible plasmid. Whole-cell lysates from *E*. *faecalis* cells grown exponentially in MH broth (supplemented with chloramphenicol for pJRG9 plasmids and erythromycin for pJLL286 plasmids and +/- 25 mM NaNO_3_) were subjected to immunoblot analysis for GlmR or RpoA (loading control). Strains and plasmids used were: Δ*glmR*, DDJ245; vector 1, pJRG9; P-*glmR*, pJLL238, vector 2, pJLL286; P_nisA_-*glmR*, pDDJ262.
(PDF)

S4 Fig Δ*glmR* mutants do not exhibit a growth defect. Bacteria were grown in MH broth and culture density monitored using a Bioscreen C plate reader. Wild type (WT) OG1, CK221 or *E*. *faecium* 1,141,733 as indicated, full line; Δ*glmR* strains as indicated, dashed line. Strains used: wild-type OG1, CK221 or *E*. *faecium* 1,141, 733; Δ*glmR*_OG1_, DDJ245; Δ*glmR*_CK221_, DDJ248, Δ*glmR*_*E*. *faecium1*,141,733_, DDJ262.
(PDF)

S5 Fig Δ*glmR* mutant does not exhibit a growth defect in semi-defined media supplemented with different carbon sources. Bacteria were grown in MM9YE without (A) or with 1% of different carbon sources, glucose (B), glycerol (C), N-Acetylglucosamine (GlcNAC, D), ribose (E), or sodium pyruvate (F). Culture density was monitored using a Bioscreen C plate reader. Wild-type OG1, full line; Δ*glmR*_OG1_ (DDJ245), dashed line.
(PDF)

S6 Fig Overexpression of GlmU, GlmM, and GlmS from a constitutive plasmid in the Δ*glmR* mutant. Whole-cell lysates from *E*. *faecalis* cells grown exponentially in MH broth (supplemented with 10 μg/ml chloramphenicol) were subjected to immunoblot analysis for GlmS, GlmM, GlmU or RpoA (loading control). Strains and plasmids used were: OG1, wild-type (WT); Δ*glmR*, DDJ245; vector, pJRG9; P-*glmU*, pJLL240, P-*glmM*, pJLL241; P-*glmS*, pJLL244.
(PDF)

S7 Fig GlmR mutants in residues predicted to be involved in uridyltransferase activity are expressed and retain the ability to bind UDP-GlcNAc. **A.** Whole-cell lysates from *E*. *faecalis* cells grown exponentially in MH broth (supplemented with erythromycin for maintenance of plasmids and +/- indicated amount of NaNO_3_) were subjected to immunoblot analysis. Strains and plasmids used were: Δ*glmR*, DDJ245; P_nisA_-*glmR*, pDDJ262; P_nisA_-*glmR*_D42-43A_, pDDJ307; P_nisA_-*glmR*_N206A_, pDDJ303. **B.** Thermal shift assay (TSA) was performed using 10 μM purified protein in the absence of compound (black line) or presence of 1 mM UDP-GlcNAc (gray line). Melting temperature profile for each protein/compound combination was assessed. UDP-GlcNAc, uridine diphosphate N-acetylglucosamine.
(PDF)

S8 Fig Multigene BLAST analysis reveals conservation of *yvcJ*-*glmR*-*yvcL* gene cluster across various Gram-positive bacterial species. A search database was generated using National Center for Biotechnology Information (NCBI) GenBank sequences of the genomes listed in the figure and Multigene BLAST, an open-source tool, used to identify a potential multigene module. Locus numbers for the *E*. *faecalis* genes in strain OG1RF are: OG1RF_10500 (*yvcJ*), OG1RF_10501 (*glmR*), OG1RF_10502 (*yvcL*).
(PDF)

S9 Fig Abundance of GlmR and YvcJ is elevated in the absence of YvcL. **A.** Expression of *yvcJ* and *glmR* genes was measured using RT-qPCR on RNA extracted from exponentially growing wild-type (WT) and Δ*yvcL* strains in MH broth. Strains used: wild-type, OG1; Δ*yvcL*, DDJ260. **B.** Bacteria were grown to exponential phase in MH broth. Whole-cell lysates were subjected to SDS-PAGE and protein expression assessed via immunoblot analysis for GlmR, YvcL, or RpoA (loading control). **C.** Bacteria were grown to exponential phase in MH broth. Whole-cell lysates were subjected to SDS-PAGE and protein expression assessed via immunoblot analysis for YvcJ or RpoA (loading control). **D.** Quantification of abundance of each protein, from panels B and C, normalized to total protein in each lane using four biological replicates. ****, p < 0.0001 determined via *t* test. *ns*, p > 0.05. Strains used were: wild-type, OG1; Δ*glmR*, DDJ245, Δ*yvcL*, DDJ260; Δ*yvcJ*, DDJ326.
(PDF)

S10 Fig Abundance of YvcJ and YvcL upon induction of expression using 5 mM nitrate. Whole-cell lysates from *E*. *faecalis* cells grown exponentially in MH broth (supplemented with erythromycin for maintenance of plasmids and +/- 5 mM NaNO_3_) were subjected to immunoblot analysis. Strains and plasmids used were: wild-type (WT), OG1; Δ*glmR*, DDJ245; Δ*yvcL*, DDJ260; Δ*yvcJ*, DDJ326; vector, pJLL286; P_nisA_-*glmR*, pDDJ262; P_nisA_-*yvcL*, pDDJ271; P_nisA_-*yvcJ*, pDDJ269.
(PDF)

S11 Fig MurAA abundance is not altered in the Δ(*yvcJ*-*glmR*) double mutant. Whole-cell lysates from *E*. *faecalis* cells grown exponentially in MH broth were subjected to immunoblot analysis. Quantification of abundance of MurAA normalized to total protein in each lane was done from three biological replicates. Wild-type (WT), OG1; Δ(*yvcJ*-*glmR*), DDJ338. RpoA is a loading control.
(PDF)

S12 Fig Alignment of GlmR proteins from *E*. *faecalis* and *B*. *subtilis*. Sequences of the proteins (*E*. *faecalis* GlmR accession AEA93188; *B*. *subtilis* GlmR accession NP_391356) were aligned and compared using a Web version of ClustalOmega. (*) signifies conserved residues.
(PDF)

S13 Fig YvcJ_K18A_ and GlmR expression is unaltered in the *yvcJ*_K18A_ mutant. Whole-cell lysates from *E*. *faecalis* cells grown exponentially in MH broth were subjected to immunoblot analysis. Quantification of abundance of YvcJ and GlmR normalized to total protein in each lane was done from two biological replicates. Strains used were: WT, OG1; Δ*yvcJ*, DDJ326; *yvcJ*_K18A_, DDJ446. RpoA is the loading control.
(PDF)

S14 Fig Images used for quantitation in Fig 3. (PDF)

S15 Fig Images used for quantitation in S9 Fig. (PDF)

S16 Fig Images used for quantitation in S11 Fig. (PDF)

S17 Fig Images used for quantitation in S13 Fig. (PDF)

## References

[1] Murray BE. 1990. The life and times of the Enterococcus. Clin Microbiol Rev 3:46–65. doi: 10.1128/CMR.3.1.46 2404568 PMC358140

[2] Tannock GW. 2002. Analysis of the intestinal microflora using molecular methods. Eur J Clin Nutr 56 Suppl 4:S44–9. 10.1038/sj.ejcn.1601661 12556947

[3] Kristich CJ, Rice LB, Arias CA. 2014. Enterococcal infection-treatment and antibiotic resistance, In: Gilmore MS, Clewell DB, Ike Y, Shankar N, editors Enterococci: from commensals to leading causes of drug resistant infection. Massachusetts Eye and Ear Infirmary, Boston, MA. 24649510

[4] Hollenbeck BL, Rice LB. 2012. Intrinsic and acquired resistance mechanisms in enterococcus. Virulence 3:421–33. doi: 10.4161/viru.21282 23076243 PMC3485979

[5] Weiner LM, Webb AK, Limbago B, Dudeck MA, Patel J, Kallen AJ, Edwards JR, Sievert DM. 2016. Antimicrobial-Resistant Pathogens Associated With Healthcare-Associated Infections: Summary of Data Reported to the National Healthcare Safety Network at the Centers for Disease Control and Prevention, 2011–2014. Infect Control Hosp Epidemiol 37:1288–1301. doi: 10.1017/ice.2016.174 27573805 PMC6857725

[6] Brinkwirth S, Ayobami O, Eckmanns T, Markwart R. 2021. Hospital-acquired infections caused by enterococci: a systematic review and meta-analysis, WHO European Region, 1 January 2010 to 4 February 2020. Euro Surveill 26. 10.2807/1560-7917.ES.2021.26.45.2001628 PMC8646982 34763754

[7] Macheboeuf P, Contreras-Martel C, Job V, Dideberg O, Dessen A. 2006. Penicillin binding proteins: key players in bacterial cell cycle and drug resistance processes. FEMS Microbiol Rev 30:673–91. doi: 10.1111/j.1574-6976.2006.00024.x 16911039

[8] Zapun A, Contreras-Martel C, Vernet T. 2008. Penicillin-binding proteins and beta-lactam resistance. FEMS Microbiol Rev 32:361–85. doi: 10.1111/j.1574-6976.2007.00095.x 18248419

[9] Sauvage E, Kerff F, Terrak M, Ayala JA, Charlier P. 2008. The penicillin-binding proteins: structure and role in peptidoglycan biosynthesis. FEMS Microbiol Rev 32:234–58. doi: 10.1111/j.1574-6976.2008.00105.x 18266856

[10] CDC. 2019. Antibiotic Resistance Threats in the United States, 2019. U.S. Department of Health and Human Services, CDC, Atlanta, GA.

[11] Kramer TS, Remschmidt C, Werner S, Behnke M, Schwab F, Werner G, Gastmeier P, Leistner R. 2018. The importance of adjusting for enterococcus species when assessing the burden of vancomycin resistance: a cohort study including over 1000 cases of enterococcal bloodstream infections. Antimicrob Resist Infect Control 7:133. doi: 10.1186/s13756-018-0419-9 30459945 PMC6234683

[12] Miller WR, Munita JM, Arias CA. 2014. Mechanisms of antibiotic resistance in enterococci. Expert Rev Anti Infect Ther 12:1221–36. doi: 10.1586/14787210.2014.956092 25199988 PMC4433168

[13] Miller WR, Murray BE, Rice LB, Arias CA. 2016. Vancomycin-Resistant Enterococci: Therapeutic Challenges in the 21st Century. Infect Dis Clin North Am 30:415–439. doi: 10.1016/j.idc.2016.02.006 27208766

[14] Carmeli Y, Eliopoulos GM, Samore MH. 2002. Antecedent Treatment with Different Antibiotic Agents as a Risk Factor for Vancomycin Resistant Enterococcus. Emerg Infect Dis 8:802–7. doi: 10.3201/eid0808.010418 12141965 PMC2732508

[15] Shepard BD, Gilmore MS. 2002. Antibiotic-resistant enterococci: the mechanisms and dynamics of drug introduction and resistance. Microbes Infect 4:215–224. doi: 10.1016/s1286-4579(01)01530-1 11880055

[16] Brandl K, Plitas G, Mihu CN, Ubeda C, Jia T, Fleisher M, Schnabl B, DeMatteo RP, Pamer EG. 2008. Vancomycin-resistant enterococci exploit antibiotic-induced innate immune deficits. Nature 455:804–7. doi: 10.1038/nature07250 18724361 PMC2663337

[17] Donskey CJ, Chowdhry TK, Hecker MT, Hoyen CK, Hanrahan JA, Hujer AM, Hutton-Thomas RA, Whalen CC, Bonomo RA, Rice LB. 2000. Effect of antibiotic therapy on the density of vancomycin-resistant enterococci in the stool of colonized patients. N Engl J Med 343:1925–32. doi: 10.1056/NEJM200012283432604 11136263 PMC4370337

[18] Ubeda C, Taur Y, Jenq RR, Equinda MJ, Son T, Samstein M, Viale A, Socci ND, van den Brink MR, Kamboj M, Pamer EG. 2010. Vancomycin-resistant Enterococcus domination of intestinal microbiota is enabled by antibiotic treatment in mice and precedes bloodstream invasion in humans. J Clin Invest 120:4332–41. doi: 10.1172/JCI43918 21099116 PMC2993598

[19] Chakraborty R, Lam V, Kommineni S, Stromich J, Hayward M, Kristich CJ, Salzman NH. 2018. Ceftriaxone Administration Disrupts Intestinal Homeostasis, Mediating Noninflammatory Proliferation and Dissemination of Commensal Enterococci. Infect Immun 86:e00674–18. doi: 10.1128/IAI.00674-18 30224553 PMC6246901

[20] Mascari CA, Djorić D, Little JL, Kristich CJ. 2022. Use of an Interspecies Chimeric Receptor for Inducible Gene Expression Reveals that Metabolic Flux through the Peptidoglycan Biosynthesis Pathway is an Important Driver of Cephalosporin Resistance in Enterococcus faecalis. J Bacteriol 204:e0060221. doi: 10.1128/jb.00602-21 35258319 PMC9017299

[21] Foulquier E, Galinier A. 2017. YvcK, a protein required for cell wall integrity and optimal carbon source utilization, binds uridine diphosphate-sugars. Sci Rep 7:4139. doi: 10.1038/s41598-017-04064-2 28646159 PMC5482804

[22] Gorke B, Foulquier E, Galinier A. 2005. YvcK of Bacillus subtilis is required for a normal cell shape and for growth on Krebs cycle intermediates and substrates of the pentose phosphate pathway. Microbiology (Reading) 151:3777–3791. doi: 10.1099/mic.0.28172-0 16272399

[23] Forouhar F, Abashidze M, Xu H, Grochowski LL, Seetharaman J, Hussain M, Kuzin A, Chen Y, Zhou W, Xiao R, Acton TB, Montelione GT, Galinier A, White RH, Tong L. 2008. Molecular insights into the biosynthesis of the F420 coenzyme. J Biol Chem 283:11832–40. doi: 10.1074/jbc.M710352200 18252724 PMC2431047

[24] Foulquier E, Pompeo F, Bernadac A, Espinosa L, Galinier A. 2011. The YvcK protein is required for morphogenesis via localization of PBP1 under gluconeogenic growth conditions in Bacillus subtilis. Mol Microbiol 80:309–18. doi: 10.1111/j.1365-2958.2011.07587.x 21320184

[25] Foulquier E, Pompeo F, Freton C, Cordier B, Grangeasse C, Galinier A. 2014. PrkC-mediated phosphorylation of overexpressed YvcK protein regulates PBP1 protein localization in Bacillus subtilis mreB mutant cells. J Biol Chem 289:23662–9. doi: 10.1074/jbc.M114.562496 25012659 PMC4156092

[26] Pensinger DA, Boldon KM, Chen GY, Vincent WJ, Sherman K, Xiong M, Schaenzer AJ, Forster ER, Coers J, Striker R, Sauer JD. 2016. The Listeria monocytogenes PASTA Kinase PrkA and Its Substrate YvcK Are Required for Cell Wall Homeostasis, Metabolism, and Virulence. PLoS Pathog 12:e1006001. doi: 10.1371/journal.ppat.1006001 27806131 PMC5091766

[27] Patel V, Wu Q, Chandrangsu P, Helmann JD. 2018. A metabolic checkpoint protein GlmR is important for diverting carbon into peptidoglycan biosynthesis in Bacillus subtilis. PLoS Genet 14:e1007689. doi: 10.1371/journal.pgen.1007689 30248093 PMC6171935

[28] Pensinger DA, Gutierrez KV, Smith HB, Vincent WJB, Stevenson DS, Black KA, Perez-Medina KM, Dillard JP, Rhee KY, Amador-Noguez D, Huynh TN, Sauer J-D. 2023. Listeria monocytogenes GlmR Is an Accessory Uridyltransferase Essential for Cytosolic Survival and Virulence. mBio 14:e00073–23. doi: 10.1128/mbio.00073-23 36939339 PMC10128056

[29] Minton NE, Djoric D, Little JL, Kristich CJ. 2022. GpsB Promotes PASTA Kinase Signaling and Cephalosporin Resistance in Enterococcus faecalis. Journal of Bacteriology 204. doi: 10.1128/jb.00304-22 36094306 PMC9578390

[30] Hall CL, Tschannen M, Worthey EA, Kristich CJ. 2013. IreB, a Ser/Thr kinase substrate, influences antimicrobial resistance in Enterococcus faecalis. Antimicrob Agents Chemother 57:6179–86. doi: 10.1128/AAC.01472-13 24080657 PMC3837872

[31] Foulquier E, Pompeo F, Byrne D, Fierobe HP, Galinier A. 2020. Uridine diphosphate N-acetylglucosamine orchestrates the interaction of GlmR with either YvcJ or GlmS in Bacillus subtilis. Sci Rep 10:15938. doi: 10.1038/s41598-020-72854-2 32994436 PMC7525490

[32] Medema MH, Takano E, Breitling R. 2013. Detecting sequence homology at the gene cluster level with MultiGeneBlast. Mol Biol Evol 30:1218–23. doi: 10.1093/molbev/mst025 23412913 PMC3670737

[33] Arntzen MO, Karlskas IL, Skaugen M, Eijsink VG, Mathiesen G. 2015. Proteomic Investigation of the Response of Enterococcus faecalis V583 when Cultivated in Urine. PLoS One 10:e0126694. doi: 10.1371/journal.pone.0126694 25915650 PMC4411035

[34] Michaux C, Hansen EE, Jenniches L, Gerovac M, Barquist L, Vogel J. 2020. Single-Nucleotide RNA Maps for the Two Major Nosocomial Pathogens Enterococcus faecalis and Enterococcus faecium. Front Cell Infect Microbiol 10:600325. doi: 10.3389/fcimb.2020.600325 33324581 PMC7724050

[35] Surdova K, Gamba P, Claessen D, Siersma T, Jonker MJ, Errington J, Hamoen LW. 2013. The conserved DNA-binding protein WhiA is involved in cell division in Bacillus subtilis. J Bacteriol 195:5450–60. doi: 10.1128/JB.00507-13 24097947 PMC3889613

[36] Kaiser BK, Stoddard BL. 2011. DNA recognition and transcriptional regulation by the WhiA sporulation factor. Sci Rep 1:156. doi: 10.1038/srep00156 22355671 PMC3240954

[37] Knizewski L, Ginalski K. 2007. Bacterial DUF199/COG1481 proteins including sporulation regulator WhiA are distant homologs of LAGLIDADG homing endonucleases that retained only DNA binding. Cell Cycle 6:1666–70. doi: 10.4161/cc.6.13.4471 17603302

[38] Comenge Y, Quintiliani R Jr., Li L, Dubost L, Brouard JP, Hugonnet JE, Arthur M. 2003. The CroRS two-component regulatory system is required for intrinsic beta-lactam resistance in Enterococcus faecalis. J Bacteriol 185:7184–92. doi: 10.1128/JB.185.24.7184-7192.2003 14645279 PMC296236

[39] Kellogg SL, Kristich CJ. 2018. Convergence of PASTA Kinase and Two-Component Signaling in Response to Cell Wall Stress in Enterococcus faecalis. J Bacteriol 200:e00086–18. doi: 10.1128/JB.00086-18 29632091 PMC5971478

[40] Kellogg SL, Little JL, Hoff JS, Kristich CJ. 2017. Requirement of the CroRS Two-Component System for Resistance to Cell Wall-Targeting Antimicrobials in Enterococcus faecium. Antimicrob Agents Chemother 61. doi: 10.1128/AAC.02461-16 28223383 PMC5404561

[41] Mascari CA, Little JL, Kristich CJ. 2023. PASTA-kinase-mediated signaling drives accumulation of the peptidoglycan synthesis protein MurAA to promote cephalosporin resistance in Enterococcus faecalis. Mol Microbiol 120:811–829. doi: 10.1111/mmi.15150 37688380 PMC10872757

[42] Kristich CJ, Djoric D, Little JL. 2014. Genetic basis for vancomycin-enhanced cephalosporin susceptibility in vancomycin-resistant enterococci revealed using counterselection with dominant-negative thymidylate synthase. Antimicrob Agents Chemother 58:1556–64. doi: 10.1128/AAC.02001-13 24366749 PMC3957902

[43] Gibson DG, Young L, Chuang RY, Venter JC, Hutchison CA 3rd, Smith HO. 2009. Enzymatic assembly of DNA molecules up to several hundred kilobases. Nat Methods 6:343–5. doi: 10.1038/nmeth.1318 19363495

[44] Langmead B, Salzberg SL. 2012. Fast gapped-read alignment with Bowtie 2. Nat Methods 9:357–9. doi: 10.1038/nmeth.1923 22388286 PMC3322381

[45] Li H, Handsaker B, Wysoker A, Fennell T, Ruan J, Homer N, Marth G, Abecasis G, Durbin R, Genome Project Data Processing S. 2009. The Sequence Alignment/Map format and SAMtools. Bioinformatics 25:2078–9. doi: 10.1093/bioinformatics/btp352 19505943 PMC2723002

[46] Li H. 2011. A statistical framework for SNP calling, mutation discovery, association mapping and population genetical parameter estimation from sequencing data. Bioinformatics 27:2987–93. doi: 10.1093/bioinformatics/btr509 21903627 PMC3198575

[47] Liao Y, Smyth GK, Shi W. 2014. featureCounts: an efficient general purpose program for assigning sequence reads to genomic features. Bioinformatics 30:923–30. doi: 10.1093/bioinformatics/btt656 24227677

[48] RCoreTeam. 2018. R: A language and environment for statistical computing. https://www.R-project.org/. https://www.R-project.org/. Accessed

[49] RStudioTeam. 2015. RStudio: Integrated Development for R. http://www.rstudio.com/.

[50] Love MI, Huber W, Anders S. 2014. Moderated estimation of fold change and dispersion for RNA-seq data with DESeq2. Genome Biol 15:550. doi: 10.1186/s13059-014-0550-8 25516281 PMC4302049

