# Supplementary material for: Reciprocal regulation of enterococcal cephalosporin resistance by products of the autoregulated yvcJ-glmR-yvcL operon enhances fitness during cephalosporin exposure
Source: PLoS Genet. 2024 Mar 21;20(3):e1011215. doi: 10.1371/journal.pgen.1011215 (PMC10986989; doi:10.1371/journal.pgen.1011215)
Supplement: S2 Table — (DOCX) [file pgen.1011215.s002.docx]

**S2 Table.** **Complementation with *E. faecalis* GlmR from a plasmid**.

|  | **MIC^a^_ceftx_ (μg/ml)** |
| --- | --- |
| WT_OG1_ (vector) | 32 |
| WT_OG1_ (P-*glmR*) | 512 |
| Δ*glmR*_OG1_ (vector) | 8 |
| Δ*glmR*_OG1_ (P-*glmR*) | 512 |
| WT_CK221_ (vector) | 512 |
| WT_CK221_ (P-*glmR*) | 2048 |
| Δ*glmR*_CK221_ (vector) | 8 |
| Δ*glmR*_CK221_ (P-*glmR*) | 1024 |
| WT_Efm_ (vector) | 64 |
| WT_Efm_ (P-*glmR*) | 1024 |
| Δ*glmR*_Efm_ (vector) | 4 |
| Δ*glmR*_Efm_ (P-*glmR*) | 256 |

^a^Median minimal inhibitory concentrations for ceftriaxone (MIC_ceftx_) determined in MH broth after a 24 h incubation at 37 °C, from a minimum of three independent experiments. Strains were: Wild-type (WT) *E. faecalis* (OG1 or CK221); wild-type *E. faecium*, WT_Efm_ (1, 141, 733); Δ*glmR*_OG1_, DDJ245; Δ*glmR*_CK221_, DDJ248, Δ*glmR*_Efm_, DDJ262. Plasmids were: vector, pJRG9; P-*glmR*, pJLL238.
