# Supplementary material for: Reciprocal regulation of enterococcal cephalosporin resistance by products of the autoregulated yvcJ-glmR-yvcL operon enhances fitness during cephalosporin exposure
Source: PLoS Genet. 2024 Mar 21;20(3):e1011215. doi: 10.1371/journal.pgen.1011215 (PMC10986989; doi:10.1371/journal.pgen.1011215)
Supplement: S3 Table — (DOCX) [file pgen.1011215.s003.docx]

**S3 Table.** **Cross-complementation of cephalosporin resistance of *E. faecalis* mutants using *E. faecium* genes**.

| **Strain** | **MIC^a^_ceftx_ (µg/ml)** |
| --- | --- |
| WT (vector) | 64 |
| WT (P-*glmR*_Efs_) | 512 |
| WT (P-*glmR*_Efm_) | 2048 |
| WT (P-*yvcJ*_Efs_) | 32 |
| WT (P-*yvcJ*_Efm_) | 64 |
| WT (P-*yvcL*_Efs_) | 32 |
| WT (P-*yvcL*_Efm_) | 64 |
| Δ*glmR* (vector) | 8 |
| Δ*glmR* (P-*glmR*_Efs_) | 512 |
| Δ*glmR* (P-*glmR*_Efm_) | 2048 |
| Δ*yvcJ*  (vector) | 512 |
| Δ*yvcJ*  (P-*yvcJ*_Efs_) | 32 |
| Δ*yvcJ* (P-*yvcJ*_Efm_) | 64 |
| Δ*yvcL*  (vector) | 256 |
| Δ*yvcL*  (P-*yvcL*_Efs_) | 32 |
| Δ*yvcL* (P-*yvcL*_Efm_) | 64 |

^a^Median minimal inhibitory concentrations for ceftriaxone (MIC_ceftx_) determined in MH broth (supplemented with Cm 10 μg/ml for plasmid maintenance) after 24 h incubation at 37 °C, from a minimum of three independent experiments.
Strains were: Wild-type, *E. faecalis* OG1; Δ*glmR*, DDJ245, Δ*yvcJ*, DDJ326; Δ*yvcL*, DDJ260. Plasmids were: vector, pJRG9; P-*glmR_Efs_*, pJLL238; P-*glmR_Efm_*, pDDJ368; P-*yvcJ_Efs_*, pDDJ276; P-*yvcJ_Efm_*, pDDJ366; P-*yvcL_Efs_*, pDDJ264; P-*yvcL_Efm_*, pDDJ367. Efs, *E. faecalis*; Efm, *E. faecium*.
