# Supplementary material for: Reciprocal regulation of enterococcal cephalosporin resistance by products of the autoregulated yvcJ-glmR-yvcL operon enhances fitness during cephalosporin exposure
Source: PLoS Genet. 2024 Mar 21;20(3):e1011215. doi: 10.1371/journal.pgen.1011215 (PMC10986989; doi:10.1371/journal.pgen.1011215)
Supplement: S4 Table — (DOCX) [file pgen.1011215.s004.docx]

**S4 Table.** **Supplementation with N-acetylglucosamine does not enhance ceftriaxone resistance.**

**MIC^a^_ceftx_ (µg/ml)**

| Strain |  |  |
| --- | --- | --- |
|  | No GlcNAc^b^ | 1 % GlcNAc^b^ |
| Wild-type | 64 | 64 |
| Δ*glmR* | 8 | 8 |

^a^Median minimal inhibitory concentrations for ceftriaxone (MIC_ceftx_) determined in MH broth (supplemented as indicated) after 24 h incubation at 37 °C, from a minimum of three independent experiments.
^b^GlcNAc, N-Acetylglucosamine.
Strains were: Wild-type, *E. faecalis* OG1; Δ*glmR*, DDJ245.
