# Supplementary material for: Reciprocal regulation of enterococcal cephalosporin resistance by products of the autoregulated yvcJ-glmR-yvcL operon enhances fitness during cephalosporin exposure
Source: PLoS Genet. 2024 Mar 21;20(3):e1011215. doi: 10.1371/journal.pgen.1011215 (PMC10986989; doi:10.1371/journal.pgen.1011215)
Supplement: S5 Table — (DOCX) [file pgen.1011215.s005.docx]

**S5 Table.** **Overexpression of GlmS, GlmM, or GlmU does not enhance ceftriaxone resistance of Δ*glmR* mutant**

| **Strain** | **MIC^a^_ceftx_ (µg/ml)** |
| --- | --- |
| WT (vector) | 32 |
| Δ*glmR* (vector) | 8 |
| Δ*glmR* (P-*glmS*) | 8 |
| Δ*glmR* (P-*glmM*) | 8 |
| Δ*glmR* (P-*glmU*) | 8 |

^a^Median minimal inhibitory concentrations for ceftriaxone (MIC_ceftx_) determined in MH broth (supplemented with Cm 10 μg/ml for plasmid maintenance) after 24 h incubation at 37 °C, from a minimum of three independent experiments.
Strains were: Wild-type (WT), *E. faecalis* OG1; Δ*glmR*, DDJ245. Plasmids were: vector, pJRG9; P-*glmU*, pJLL240; P-*glmS*, pJLL244; P-*glmM*, pJLL241.
