# Supplementary material for: Reciprocal regulation of enterococcal cephalosporin resistance by products of the autoregulated yvcJ-glmR-yvcL operon enhances fitness during cephalosporin exposure
Source: PLoS Genet. 2024 Mar 21;20(3):e1011215. doi: 10.1371/journal.pgen.1011215 (PMC10986989; doi:10.1371/journal.pgen.1011215)
Supplement: S6 Table — (DOCX) [file pgen.1011215.s006.docx]

**S6 Table.** **Differentially expressed genes identified from RNASeq analysis of the *yvcL* deletion mutant reveals regulation of *yvcJ* and *glmR*.**

| Gene locus | Annotation in OG1RF | log_2_FoldChange mutant vs. wild-type | adj p-value | qRT-PCR mutant vs. wild-type  (fold change ± SE^a^) |
| --- | --- | --- | --- | --- |
| OG1RF_10234 | 6-phospho-beta-glucosidase | -1.0588 | 0.000893 | ND^b^ |
| OG1RF_10235 | PTS family lactose-N,N'-diacetylchitobiose-beta-glucoside (lac) porter component IIBC | -1.54454 | 0.000523 | ND^b^ |
| OG1RF_10500 | (rapZ or yvcJ) ATP-binding protein | 1.649559 | 7.70E-75 | 2.4 ± 0.07 |
| OG1RF_10501 | Hypothetical protein (yvcK or glmR) | 1.790827 | 4.53E-96 | 1.5 ± 0.06 |
| OG1RF_10502 | Hypothetical protein (whiA or yvcL) | -2.97254 | 1.30E-167 | not detected |
| OG1RF_10503 | PEP phosphonomutase family protein | 2.546418 | 9.56E-73 | 7.4 ± 0.4 |
| OG1RF_10745 | PTS system transporter subunit I | -1.33198 | 7.79E-07 | ND^b^ |
| OG1RF_10746 | PTS family lactose/cellobiose porter component IIC | -1.06514 | 0.007669 | ND^b^ |
| OG1RF_10750 | PTS system transporter subunit I | -1.09682 | 1.12E-06 | ND^b^ |
| OG1RF_10751 | PTS family lactose/cellobiose porter component IIA | -1.00836 | 9.42E-06 | ND^b^ |
| OG1RF_11182 | molybdenum cofactor biosynthesis protein A | -1.12733 | 0.009359 | ND^b^ |
| OG1RF_11372 | 3-methyl-2-oxobutanoate dehydrogenase | -1.26511 | 0.004026 | ND^b^ |
| OG1RF_11373 | dihydrolipoyl dehydrogenase | -1.4099 | 0.038034 | ND^b^ |
| OG1RF_11374 | butyrate kinase | -1.45719 | 9.33E-05 | ND^b^ |
| OG1RF_11375 | branched-chain phosphotransacylase | -1.77189 | 0.000523 | ND^b^ |
| OG1RF_12404 | D-isomer specific 2-hydroxyacid dehydrogenase | -1.21594 | 3.26E-05 | ND^b^ |
| OG1RF_12405 | 6-phosphogluconate dehydrogenase | -1.42386 | 2.24E-06 | ND^b^ |
| OG1RF_12477 | PTS family ascorbate porter, IIB component | -1.13197 | 0.007226 | ND^b^ |
| OG1RF_12555 | SDR family dehydrogenase | -1.44822 | 3.93E-07 | ND^b^ |
| OG1RF_12572 | citrate transporter | -1.11927 | 3.55E-10 | ND^b^ |

^a^SE, Standard error. ^b^ND, not determined.
