# Supplementary material for: Reciprocal regulation of enterococcal cephalosporin resistance by products of the autoregulated yvcJ-glmR-yvcL operon enhances fitness during cephalosporin exposure
Source: PLoS Genet. 2024 Mar 21;20(3):e1011215. doi: 10.1371/journal.pgen.1011215 (PMC10986989; doi:10.1371/journal.pgen.1011215)
Supplement: S7 Table — (DOCX) [file pgen.1011215.s007.docx]

**S7 Table.** **Complementation of Δ*yvcJ* and Δ*yvcL* mutants rescues ceftriaxone resistance to wild-type level.**

| **Strain** | **MIC^a^_ceftx_   no inducer** | **(µg/ml)  5 mM NaNO_3_** |
| --- | --- | --- |
| WT (vector) | 128 | 128 |
| Δ*glmR* (vector) | 8 | 8 |
| Δ*glmR* (P_nisA_-*glmR*) | 32 | 128 |
| Δ*yvcL* (vector) | 256 | 256 |
| Δ*yvcL* (P_nisA_-*yvcL*) | 256 | 128 |
| Δ*yvcJ* (vector) | 1024 | 1024 |
| Δ*yvcJ* (P_nisA_-*yvcJ*) | 512 | 64 |

^a^Median minimal inhibitory concentrations for ceftriaxone (MIC_ceftx_) determined in MH broth (supplemented with 10 μg/ml erythromycin for plasmid maintenance and indicated nitrate concentration to induce expression) after 24 h incubation at 37 °C, from a minimum of three independent experiments. Strains were: Wild-type (WT), *E. faecalis* OG1; Δ*glmR*, DDJ245; Δ*yvcL*, DDJ260; Δ*yvcJ*, DDJ326. Plasmids were: vector, pJLL286; P_nisA_-*glmR*, pDDJ262; P_nisA_-*yvcL*, pDDJ271; P_nisA_-*yvcJ*, pDDJ269.
