# Supplementary material for: Reciprocal regulation of enterococcal cephalosporin resistance by products of the autoregulated yvcJ-glmR-yvcL operon enhances fitness during cephalosporin exposure
Source: PLoS Genet. 2024 Mar 21;20(3):e1011215. doi: 10.1371/journal.pgen.1011215 (PMC10986989; doi:10.1371/journal.pgen.1011215)
Supplement: S8 Table — (DOCX) [file pgen.1011215.s008.docx]

**S8 Table.** **Ectopic expression of GlmR restores resistance of the Δ(*glmR* *yvcL*) mutant**.

| **Strain** | **MIC^a^_ceftx_ (µg/ml)** |
| --- | --- |
| Δ*yvcL* (vector) | 128 |
| Δ*yvcL* (P-*glmR*) | 512 |
| Δ*glmR* (vector) | 8 |
| Δ*glmR* (P-*glmR*) | 512 |
| Δ(*glmR-yvcL*) (vector) | 4 |
| Δ(*glmR-yvcL*) (P-*glmR*) | 512 |

^a^Median minimal inhibitory concentrations for ceftriaxone (MIC_ceftx_) determined in MH broth (supplemented with 10 μg/ml chloramphenicol for plasmid maintenance when necessary) after 24 h incubation at 37 °C, from a minimum of three independent experiments.
Strains were: Δ*ireK*, JL206; Δ*glmR* Δ*ireK,* DDJ322. Plasmids were: vector, pJRG9; P-*glmR*, pJLL238.
