# Supplementary material for: Reciprocal regulation of enterococcal cephalosporin resistance by products of the autoregulated yvcJ-glmR-yvcL operon enhances fitness during cephalosporin exposure
Source: PLoS Genet. 2024 Mar 21;20(3):e1011215. doi: 10.1371/journal.pgen.1011215 (PMC10986989; doi:10.1371/journal.pgen.1011215)
Supplement: S9 Table — (DOCX) [file pgen.1011215.s009.docx]

**S9 Table.**  Strains and plasmids used in this study

| **Strain or plasmid** | **Relevant genotype or description^a^** | **Source or reference** |
| --- | --- | --- |
| Strains |  |  |
| *E. coli* |  |  |
| TOP10 | Routine cloning host | Lab stock |
| LE392 | Host for allelic exchange plasmids | Lab stock |
| BL21 [DE3] | *E. coli* protein expression host | Lab stock |
| *E. faecalis* |  |  |
| OG1 | Wild-type reference strain (MLST 1) | *^1^* |
| DDJ245 | OG1 Δ*glmR* (ΔR7-I276) | This work |
| DDJ260 | OG1 Δ*yvcL* (ΔV7-K305) | This work |
| DDJ326 | OG1 Δ*yvcJ* (ΔL7-S246) | This work |
| DDJ332 | OG1 Δ(*glmR*-*yvcL)* | This work |
| DDJ338 | OG1 Δ(*yvcJ*-*glmR)* | This work |
| DDJ322 | OG1 Δ*glmR* Δ*ireK* | This work |
| DDJ446 | OG1 *yvcJ*_K18A_ | This work |
| JL206 | OG1 Δ*ireK* | *^2^* |
| OG1Sp | Spontaneous Sp^R^ derivative of OG1 | *^3^* |
| CK138 | Spontaneous Fa^R^ derivative of OG1 | *^4^* |
| V583 | *vanB*-containing clinical isolate (MLST 6) | *^5^* |
| CK221 | V583 Δ*ermB* (Em^S^) | *^6^* |
| DDJ248 | Δ*glmR* (ΔR7-I276) | This work |
| *E. faecium* |  |  |
| 1,141,733 | Wild-type reference strain, clinical isolate (MLST 327) | *^7^* |
| DDJ262 | 1141733 Δ*glmR* (ΔR7-I275) | This work |
|  |  |  |
| Plasmids |  |  |
| pCJK245 | *E.faecalis* allelic exchange vector (Cm^R^), thyA* counterselection | *^8^* |
| pJH086 | *E. faecalis* allelic exchange vector (Cm^R^); *pheS** counterselection | *^9^* |
| pJLL69 | pCJK218:: Δ*ireK* | *^10^* |
| pDDJ229 | pCJK245::Δ*glmR*_OG1_ | This work |
| pDDJ230 | pCJK245::Δ*glmR*_CK221_ | This work |
| pDDJ231 | pCJK245::Δ*glmR_E. faecium_* | This work |
| pDDJ261 | pJH086:: Δ*yvcL* | This work |
| pDDJ273 | pJH086:: Δ*yvcJ* | This work |
| pDDJ275 | pJH086:: Δ(*glmR-yvcL)* | This work |
| pDDJ278 | pJH086:: Δ(*yvcJ-glmR)* | This work |
| pDDJ296 | pJH086::*yvcJ*_K18A_ |  |
|  |  |  |
| pJRG9 | Enterococcal expression vector carrying constitutive P23s promoter (Cm^R^) | *^11^* |
| pJLL238 | pJRG9::*glmR* | This work |
| pJLL244 | pJRG9::*glmS* | This work |
| pJLL240 | pJRG9::*glmU* | This work |
| pJLL241 | pJRG9::*glmM* | This work |
| pDDJ264 | pJRG9::*yvcL* | This work |
| pDDJ276 | pJRG9::*yvcJ* | This work |
| pDDJ366 | pJRG9::*yvcJ* *E. faecium* | This work |
| pDDJ367 | pJRG9::*yvcL* *E. faecium* | This work |
| pDDJ368 | pJRG9::*glmR* *E. faecium* | This work |
| pJLL286 | Nitrate-inducible expression vector (Em^R^) | *^12^* |
| pDDJ262 | pJLL286 carrying P*_nisA_*::*glmR* | This work |
| pDDJ269 | pJLL286 carrying P*_nisA_*::*yvcJ* | This work |
| pDDJ271 | pJLL286 carrying P*_nisA_*::*yvcL* | This work |
| pDDJ303 | pJLL286 carrying P*_nisA_*::*glmR*_N206A_ | This work |
| pDDJ306 | pJLL286 carrying P*_nisA_*::*glmR_D42A D43A_* _N206A_ | This work |
| pDDJ307 | pJLL286 carrying P*_nisA_*::*glmR_D42A D43A_* | This work |
| pET28a::His_6_-Smt_3_ | *E. coli* protein expression vector (Kn^R^) encoding cleavable His_6_-SUMO tag | *^13^* |
| pDDJ237 | pET28a::His_6_-Smt_3_-*glmR* | This work |
| pDDJ291 | pET28a::His_6_-Smt_3_-*yvcJ* | This work |
| pDDJ292 | pET28a::His_6_-Smt_3_-*yvcL* | This work |
| pDDJ311 | pET28a::His_6_-Smt_3_-*glmR*_N206A_ | This work |
| pDDJ313 | pET28a::His_6_-Smt_3_-*glmR*_D42A D43A_ | This work |
| pDDJ331 | pET28a::His_6_-Smt_3_-*yvcJ*_K18A_ | This work |

^a^MLST, multilocus sequence type.

**SUPPLEMENTAL REFERENCES**

[1] Gold, O. G., Jordan, H. G., and van Houte, J. (1975) The Prevalence of enterococci in the human mouth and their pathogenicity in animal models *Archs oral Biol* *20*, 473-477.

[2] Labbe, B. D., and Kristich, C. J. (2017) Growth- and Stress-Induced PASTA Kinase Phosphorylation in *Enterococcus faecalis*, *J Bacteriol* *199*, e00363-00317.

[3] Kristich, C. J., Chandler, J. R., and Dunny, G. M. (2007) Development of a host-genotype-independent counterselectable marker and a high-frequency conjugative delivery system and their use in genetic analysis of *Enterococcus faecalis*, *Plasmid* *57*, 131–144.

[4] Kristich, C. J., Little, J. L., Hall, C. L., and Hoff, J. S. (2011) Reciprocal regulation of cephalosporin resistance in *Enterococcus faecalis*, *mBio* *2*, e00199-00111.

[5] Sahm, D. F., Kissinger, J., Gilmore, M. S., Murray, P. R., Mulder, R., Solliday, J., and Clarke, B. (1989) In vitro susceptibility studies of vancomycin-resistant *Enterococcus faecalis*, *Antimicrob Agents Chemother* *33*, 1588-1591.

[6] Djoric, D., and Kristich, C. J. (2017) Extracellular SalB Contributes to Intrinsic Cephalosporin Resistance and Cell Envelope Integrity in *Enterococcus faecalis*, *J Bacteriol* *199*.

[7] Palmer, K. L., Carniol, K., Manson, J. M., Heiman, D., Shea, T., Young, S., Zeng, Q., Gevers, D., Feldgarden, M., Birren, B., and Gilmore, M. S. (2010) High-quality draft genome sequences of 28 *Enterococcus* sp. isolates, *J Bacteriol* *192*, 2469-2470.

[8] Kristich, C. J., Djoric, D., and Little, J. L. (2014) Genetic basis for vancomycin-enhanced cephalosporin susceptibility in vancomycin-resistant enterococci revealed using counterselection with dominant-negative thymidylate synthase, *Antimicrob Agents Chemother* *58*, 1556-1564.

[9] Kellogg, S. L., Little, J. L., Hoff, J. S., and Kristich, C. J. (2017) Requirement of the CroRS Two-Component System for Resistance to Cell Wall-Targeting Antimicrobials in *Enterococcus faecium*, *Antimicrob Agents Chemother* *61*.

[10] Kellogg, S. L., and Kristich, C. J. (2018) Convergence of PASTA Kinase and Two-Component Signaling in Response to Cell Wall Stress in *Enterococcus faecalis*, *J Bacteriol* *200*, e00086-00018.

[11] Snyder, H., Kellogg, S. L., Skarda, L. M., Little, J. L., and Kristich, C. J. (2014) Nutritional control of antibiotic resistance via an interface between the phosphotransferase system and a two-component signaling system, *Antimicrob Agents Chemother* *58*, 957-965.

[12] Mascari, C. A., Djorić, D., Little, J. L., and Kristich, C. J. (2022) Use of an Interspecies Chimeric Receptor for Inducible Gene Expression Reveals that Metabolic Flux through the Peptidoglycan Biosynthesis Pathway is an Important Driver of Cephalosporin Resistance in *Enterococcus faecalis*, *J Bacteriol* *204*, e0060221.

[13] Hall, C. L., Lytle, B. L., Jensen, D., Hoff, J. S., Peterson, F. C., Volkman, B. F., and Kristich, C. J. (2017) Structure and Dimerization of IreB, a Negative Regulator of Cephalosporin Resistance in *Enterococcus faecalis*, *J Mol Biol* *429*, 2324-2336.
