# Supplementary material for: Reciprocal regulation of enterococcal cephalosporin resistance by products of the autoregulated yvcJ-glmR-yvcL operon enhances fitness during cephalosporin exposure
Source: PLoS Genet. 2024 Mar 21;20(3):e1011215. doi: 10.1371/journal.pgen.1011215 (PMC10986989; doi:10.1371/journal.pgen.1011215)
Supplement: S1 Fig — Expression of GlmR from the E. faecalis chromosome was compared to expression from a plasmid in both wild-type (WT) and ΔglmR mutant. Whole-cell lysates from E. faecalis cells grown exponentially in MH broth (+/- chloramphenicol for maintenance of plasmids) were subjected to immunoblot analysis for GlmR or RpoA (loading control). Strains and plasmids used were: WT, OG1; ΔglmR, DDJ245; vector, pJRG9; P-glmR, pJLL238. (PDF) [file pgen.1011215.s010.pdf]

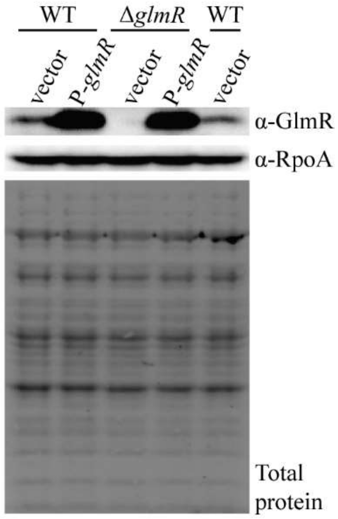

S1 Fig. GlmR is overexpressed from a plasmid. Expression of GlmR from the *E. faecalis* chromosome was compared to expression from a plasmid in both wild-type (WT) and Δ*glmR* mutant. Whole-cell lysates from *E. faecalis* cells grown exponentially in MH broth (+/- chloramphenicol for maintenance of plasmids) were subjected to immunoblot analysis for GlmR or RpoA (loading control). Strains and plasmids used were: WT, OG1; Δ*glmR*, DDJ245; vector, pJRG9; P-*glmR*, pJLL238.
