# Supplementary material for: Reciprocal regulation of enterococcal cephalosporin resistance by products of the autoregulated yvcJ-glmR-yvcL operon enhances fitness during cephalosporin exposure
Source: PLoS Genet. 2024 Mar 21;20(3):e1011215. doi: 10.1371/journal.pgen.1011215 (PMC10986989; doi:10.1371/journal.pgen.1011215)
Supplement: S2 Fig — Whole-cell lysates from E. faecalis cells grown exponentially in MH broth (+/- erythromycin for maintenance of plasmids and listed nitrate concentrations) were subjected to immunoblot analysis for GlmR or RpoA (loading control). Strains and plasmids used were: wild-type (WT), OG1; ΔglmR, DDJ245; vector, pJLL286; PnisA-glmR, pDDJ262. (PDF) [file pgen.1011215.s011.pdf]

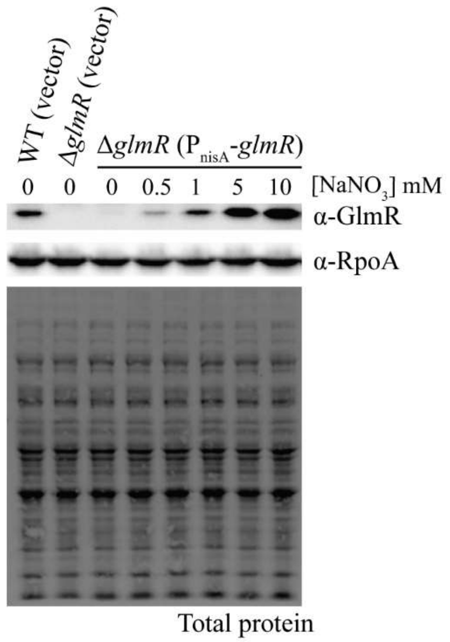

S2 Fig. Dose-dependent increase in GlmR abundance upon induction with nitrate. Whole-cell lysates from *E. faecalis* cells grown exponentially in MH broth (+/- erythromycin for maintenance of plasmids and listed nitrate concentrations) were subjected to immunoblot analysis for GlmR or RpoA (loading control). Strains and plasmids used were: wild-type (WT), OG1; Δ*glmR*, DDJ245; vector, pJLL286; P_{nisA}-*glmR*, pDDJ262.
