# Supplementary material for: Reciprocal regulation of enterococcal cephalosporin resistance by products of the autoregulated yvcJ-glmR-yvcL operon enhances fitness during cephalosporin exposure
Source: PLoS Genet. 2024 Mar 21;20(3):e1011215. doi: 10.1371/journal.pgen.1011215 (PMC10986989; doi:10.1371/journal.pgen.1011215)
Supplement: S3 Fig — Whole-cell lysates from E. faecalis cells grown exponentially in MH broth (supplemented with chloramphenicol for pJRG9 plasmids and erythromycin for pJLL286 plasmids and +/- 25 mM NaNO3) were subjected to immunoblot analysis for GlmR or RpoA (loading control). Strains and plasmids used were: ΔglmR, DDJ245; vector 1, pJRG9; P-glmR, pJLL238, vector 2, pJLL286; PnisA-glmR, pDDJ262. (PDF) [file pgen.1011215.s012.pdf]

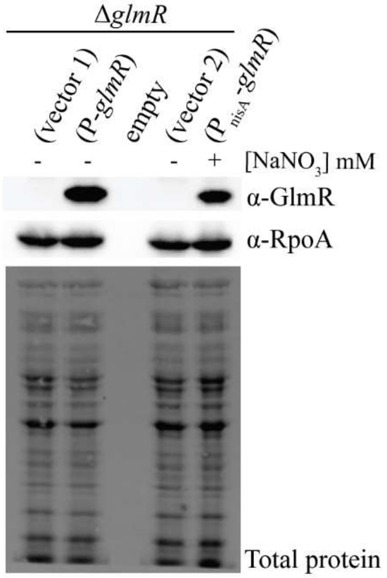

S3 Fig. Lower expression of GlmR is observed from nitrate-inducible plasmid. Whole-cell lysates from *E. faecalis* cells grown exponentially in MH broth (supplemented with chloramphenicol for pJRG9 plasmids and erythromycin for pJLL286 plasmids and +/- 25 mM $NaNO_3$) were subjected to immunoblot analysis for GlmR or RpoA (loading control). Strains and plasmids used were: Δ*glmR*, DDJ245; vector 1, pJRG9; P-*glmR*, pJLL238, vector 2, pJLL286; P_{nisA}-*glmR*, pDDJ262.
