# Supplementary material for: Reciprocal regulation of enterococcal cephalosporin resistance by products of the autoregulated yvcJ-glmR-yvcL operon enhances fitness during cephalosporin exposure
Source: PLoS Genet. 2024 Mar 21;20(3):e1011215. doi: 10.1371/journal.pgen.1011215 (PMC10986989; doi:10.1371/journal.pgen.1011215)
Supplement: S4 Fig — Bacteria were grown in MH broth and culture density monitored using a Bioscreen C plate reader. Wild type (WT) OG1, CK221 or E. faecium 1,141,733 as indicated, full line; ΔglmR strains as indicated, dashed line. Strains used: wild-type OG1, CK221 or E. faecium 1,141, 733; ΔglmROG1, DDJ245; ΔglmRCK221, DDJ248, ΔglmRE. faecium1,141,733, DDJ262. (PDF) [file pgen.1011215.s013.pdf]

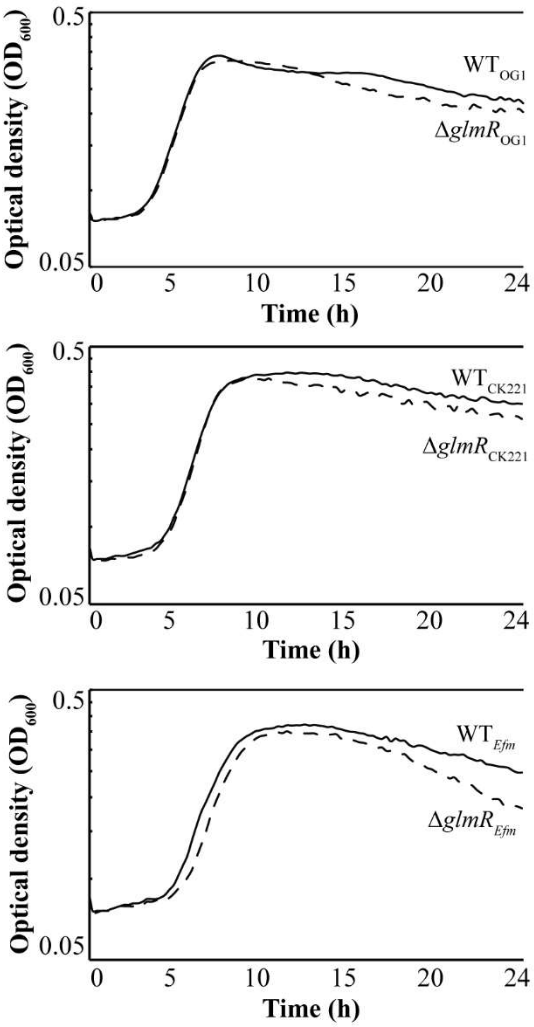

S4 Fig. Δ*glmR* mutants do not exhibit a growth defect. Bacteria were grown in MH broth and culture density monitored using a Bioscreen C plate reader. Wild type (WT) OG1, CK221 or *E. faecium* 1,141,733 as indicated, full line; Δ*glmR* strains as indicated, dashed line. Strains used: wild-type OG1, CK221 or *E. faecium* 1,141, 733; Δ*glmR*$_{\mathrm{OG1}}$, DDJ245; Δ*glmR*$_{\mathrm{CK221}}$, DDJ248; Δ*glmR*$_{E.\ faecium1,141,733}$, DDJ262.
