# Supplementary material for: Reciprocal regulation of enterococcal cephalosporin resistance by products of the autoregulated yvcJ-glmR-yvcL operon enhances fitness during cephalosporin exposure
Source: PLoS Genet. 2024 Mar 21;20(3):e1011215. doi: 10.1371/journal.pgen.1011215 (PMC10986989; doi:10.1371/journal.pgen.1011215)
Supplement: S5 Fig — Bacteria were grown in MM9YE without (A) or with 1% of different carbon sources, glucose (B), glycerol (C), N-Acetylglucosamine (GlcNAC, D), ribose (E), or sodium pyruvate (F). Culture density was monitored using a Bioscreen C plate reader. Wild-type OG1, full line; ΔglmROG1 (DDJ245), dashed line. (PDF) [file pgen.1011215.s014.pdf]

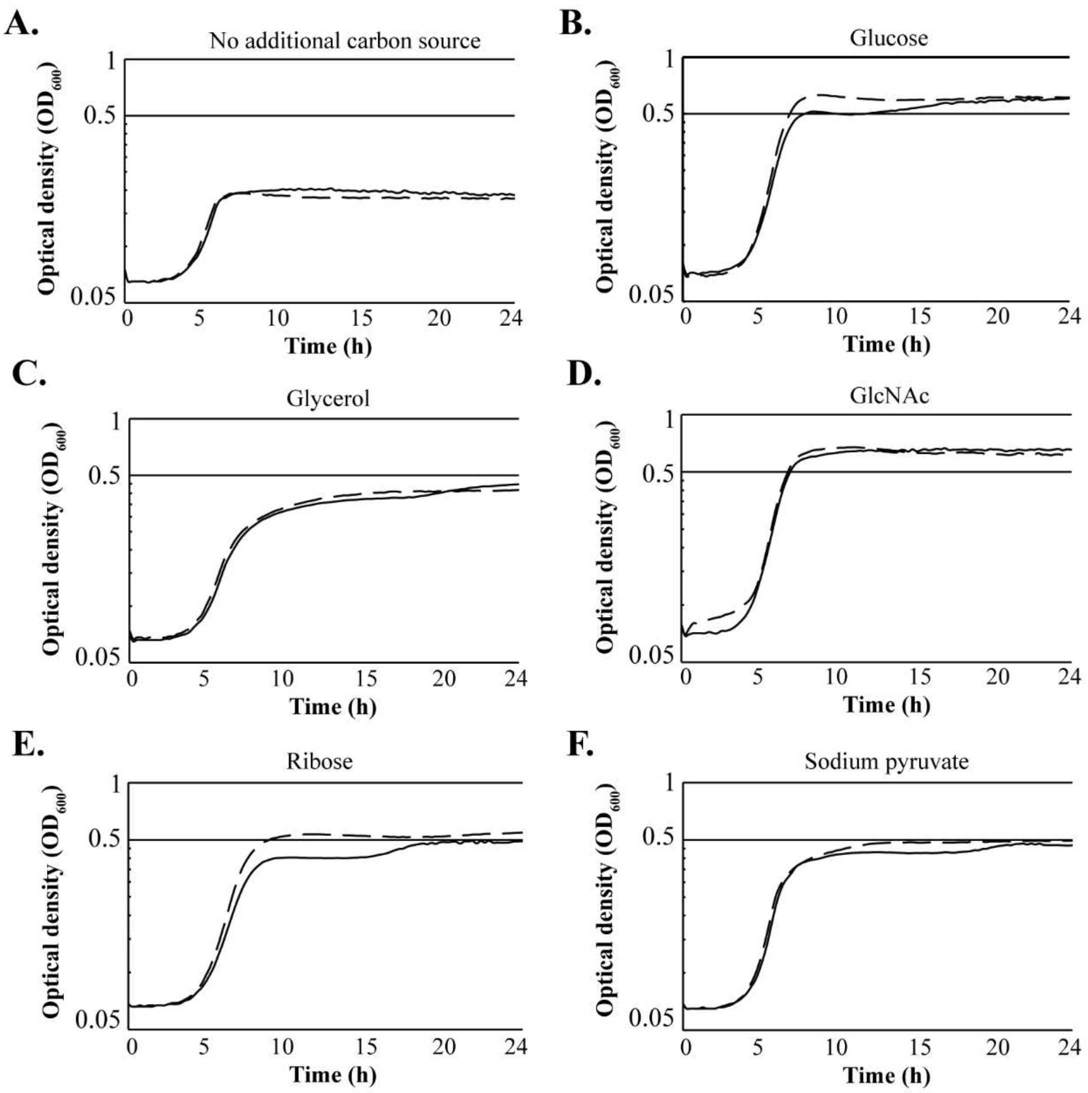

S5 Fig. Δ*glmR* mutant does not exhibit a growth defect in semi-defined media supplemented with different carbon sources. Bacteria were grown in MM9YE without (A) or with 1% of different carbon sources, glucose (B), glycerol (C), N-Acetylglucosamine (GlcNAC, D), ribose (E), or sodium pyruvate (F). Culture density was monitored using a Bioscreen C plate reader. Wild-type OG1, full line; Δ$glmR_{OG1}$ (DDJ245), dashed line.
