# Supplementary material for: Reciprocal regulation of enterococcal cephalosporin resistance by products of the autoregulated yvcJ-glmR-yvcL operon enhances fitness during cephalosporin exposure
Source: PLoS Genet. 2024 Mar 21;20(3):e1011215. doi: 10.1371/journal.pgen.1011215 (PMC10986989; doi:10.1371/journal.pgen.1011215)
Supplement: S6 Fig — Whole-cell lysates from E. faecalis cells grown exponentially in MH broth (supplemented with 10 μg/ml chloramphenicol) were subjected to immunoblot analysis for GlmS, GlmM, GlmU or RpoA (loading control). Strains and plasmids used were: OG1, wild-type (WT); ΔglmR, DDJ245; vector, pJRG9; P-glmU, pJLL240, P-glmM, pJLL241; P-glmS, pJLL244. (PDF) [file pgen.1011215.s015.pdf]

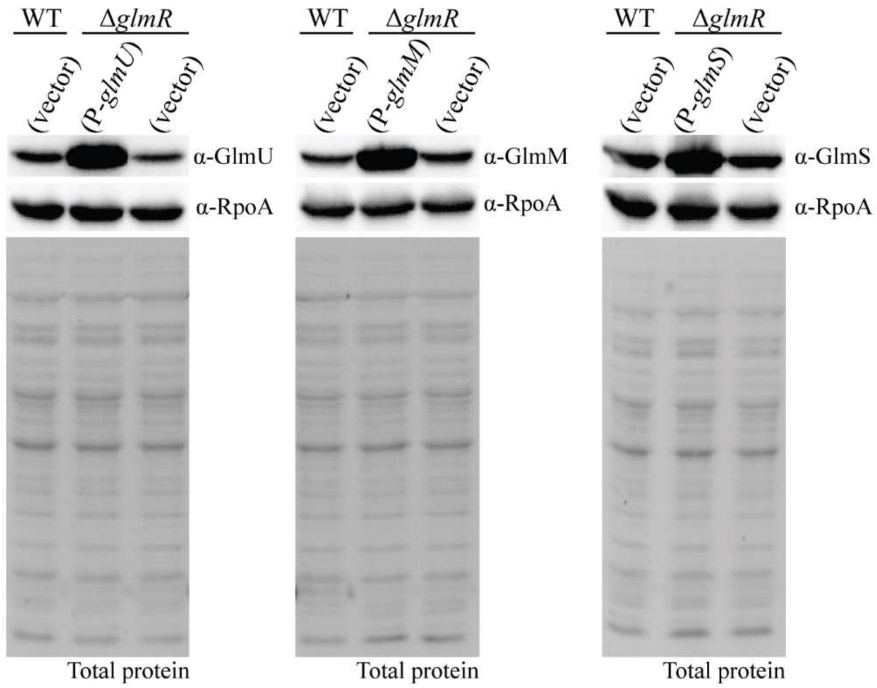

S6 Fig. Overexpression of GlmU, GlmM, and GlmS from a constitutive plasmid in the Δ*glmR* mutant. Whole-cell lysates from *E. faecalis* cells grown exponentially in MH broth (supplemented with 10 µg/ml chloramphenicol) were subjected to immunoblot analysis for GlmS, GlmM, GlmU or RpoA (loading control). Strains and plasmids used were: OG1, wild-type (WT); Δ*glmR*, DDJ245; vector, pJRG9; P-*glmU*, pJLL240, P-*glmM*, pJLL241; P-*glmS*, pJLL244.
