# Supplementary material for: Reciprocal regulation of enterococcal cephalosporin resistance by products of the autoregulated yvcJ-glmR-yvcL operon enhances fitness during cephalosporin exposure
Source: PLoS Genet. 2024 Mar 21;20(3):e1011215. doi: 10.1371/journal.pgen.1011215 (PMC10986989; doi:10.1371/journal.pgen.1011215)
Supplement: S7 Fig — A. Whole-cell lysates from E. faecalis cells grown exponentially in MH broth (supplemented with erythromycin for maintenance of plasmids and +/- indicated amount of NaNO3) were subjected to immunoblot analysis. Strains and plasmids used were: ΔglmR, DDJ245; PnisA-glmR, pDDJ262; PnisA-glmRD42-43A, pDDJ307; PnisA-glmRN206A, pDDJ303. B. Thermal shift assay (TSA) was performed using 10 μM purified protein in the absence of compound (black line) or presence of 1 mM UDP-GlcNAc (gray line). Melting temperature profile for each protein/compound combination was assessed. UDP-GlcNAc, uridine diphosphate N-acetylglucosamine. (PDF) [file pgen.1011215.s016.pdf]

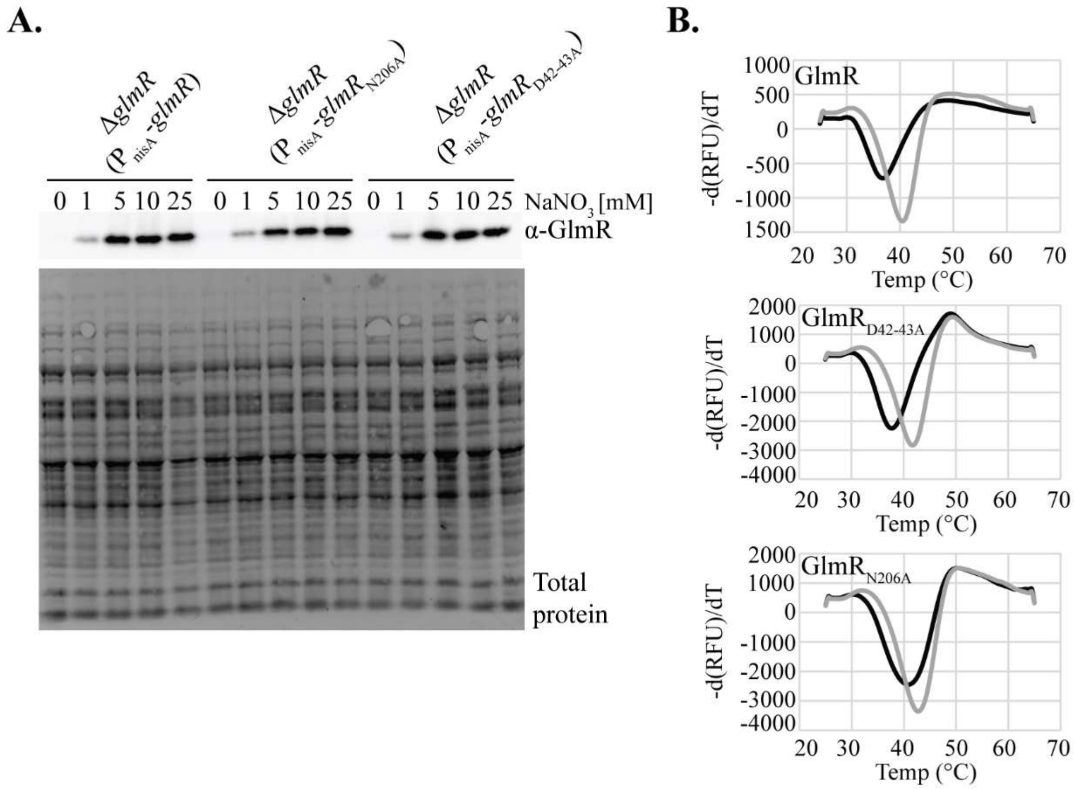

S7 Fig. GlmR mutants in residues predicted to be involved in uridyltransferase activity are expressed and retain the ability to bind UDP-GlcNAc. A. Whole-cell lysates from *E. faecalis* cells grown exponentially in MH broth (supplemented with erythromycin for maintenance of plasmids and +/- indicated amount of $NaNO_3$) were subjected to immunoblot analysis. Strains and plasmids used were: Δ*glmR*, DDJ245; P_{nisA}-*glmR*, pDDJ262; P_{nisA}-*glmR*$_{D42\text{-}43A}$, pDDJ307; P_{nisA}-*glmR*$_{N206A}$, pDDJ303. **B.** Thermal shift assay (TSA) was performed using 10 µM purified protein in the absence of compound (black line) or presence of 1 mM UDP-GlcNAc (gray line). Melting temperature profile for each protein/compound combination was assessed. UDP-GlcNAc, uridine diphosphate N-acetylglucosamine.
