# Supplementary material for: Reciprocal regulation of enterococcal cephalosporin resistance by products of the autoregulated yvcJ-glmR-yvcL operon enhances fitness during cephalosporin exposure
Source: PLoS Genet. 2024 Mar 21;20(3):e1011215. doi: 10.1371/journal.pgen.1011215 (PMC10986989; doi:10.1371/journal.pgen.1011215)
Supplement: S8 Fig — A search database was generated using National Center for Biotechnology Information (NCBI) GenBank sequences of the genomes listed in the figure and Multigene BLAST, an open-source tool, used to identify a potential multigene module. Locus numbers for the E. faecalis genes in strain OG1RF are: OG1RF_10500 (yvcJ), OG1RF_10501 (glmR), OG1RF_10502 (yvcL). (PDF) [file pgen.1011215.s017.pdf]

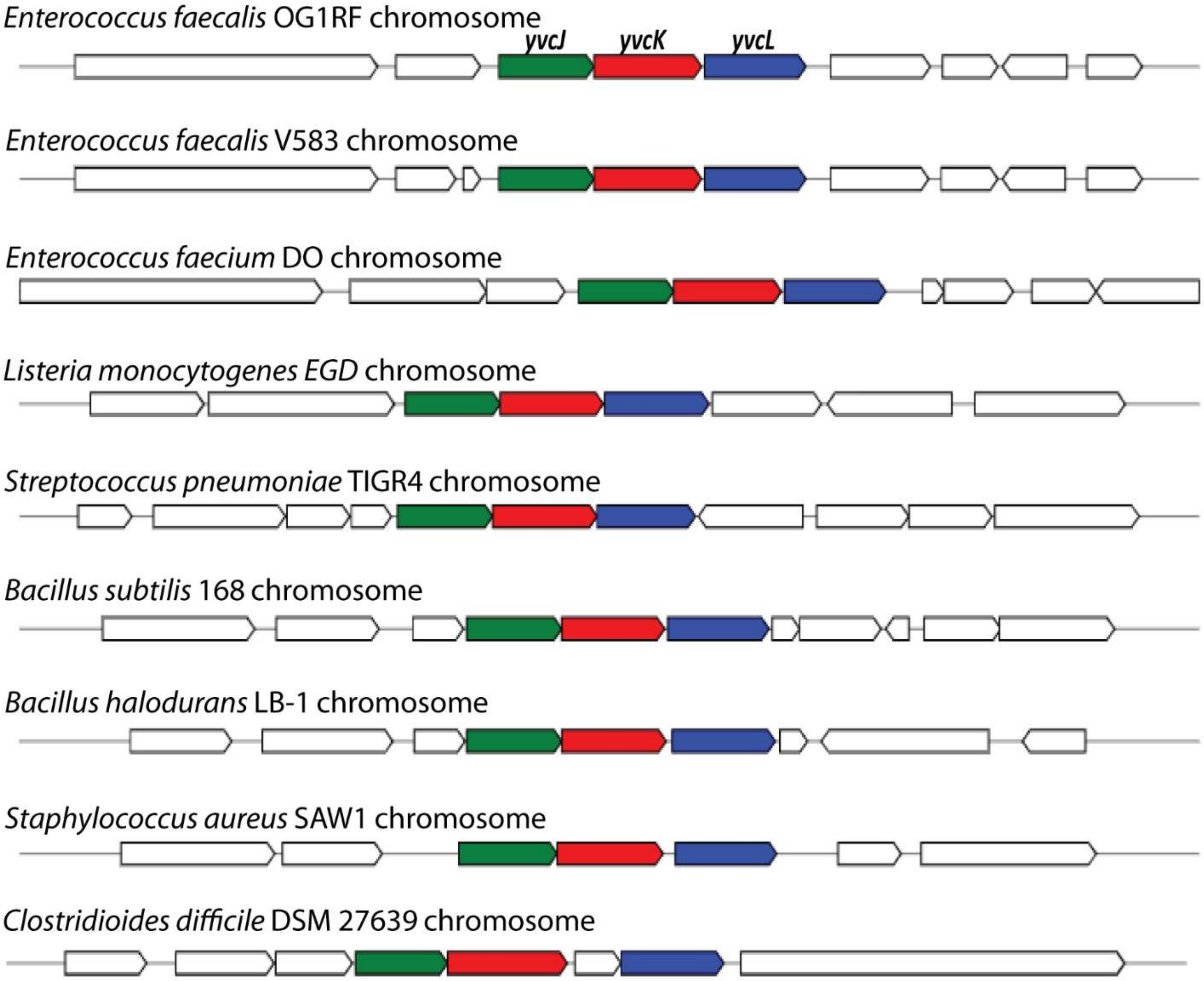

S8 Fig. Multigene BLAST analysis reveals conservation of *yvcJ-glmR-yvcL* gene cluster across various Gram-positive bacterial species. A search database was generated using National Center for Biotechnology Information (NCBI) GenBank sequences of the genomes listed in the figure and Multigene BLAST, an open-source tool, used to identify a potential multigene module. Locus numbers for the *E. faecalis* genes in strain OG1RF are: OG1RF_10500 (*yvcJ*), OG1RF_10501 (*glmR*), OG1RF_10502 (*yvcL*).
