# Supplementary material for: Reciprocal regulation of enterococcal cephalosporin resistance by products of the autoregulated yvcJ-glmR-yvcL operon enhances fitness during cephalosporin exposure
Source: PLoS Genet. 2024 Mar 21;20(3):e1011215. doi: 10.1371/journal.pgen.1011215 (PMC10986989; doi:10.1371/journal.pgen.1011215)
Supplement: S9 Fig — A. Expression of yvcJ and glmR genes was measured using RT-qPCR on RNA extracted from exponentially growing wild-type (WT) and ΔyvcL strains in MH broth. Strains used: wild-type, OG1; ΔyvcL, DDJ260. B. Bacteria were grown to exponential phase in MH broth. Whole-cell lysates were subjected to SDS-PAGE and protein expression assessed via immunoblot analysis for GlmR, YvcL, or RpoA (loading control). C. Bacteria were grown to exponential phase in MH broth. Whole-cell lysates were subjected to SDS-PAGE and protein expression assessed via immunoblot analysis for YvcJ or RpoA (loading control). D. Quantification of abundance of each protein, from panels B and C, normalized to total protein in each lane using four biological replicates. ****, p < 0.0001 determined via t test. ns, p > 0.05. Strains used were: wild-type, OG1; ΔglmR, DDJ245, ΔyvcL, DDJ260; ΔyvcJ, DDJ326. (PDF) [file pgen.1011215.s018.pdf]

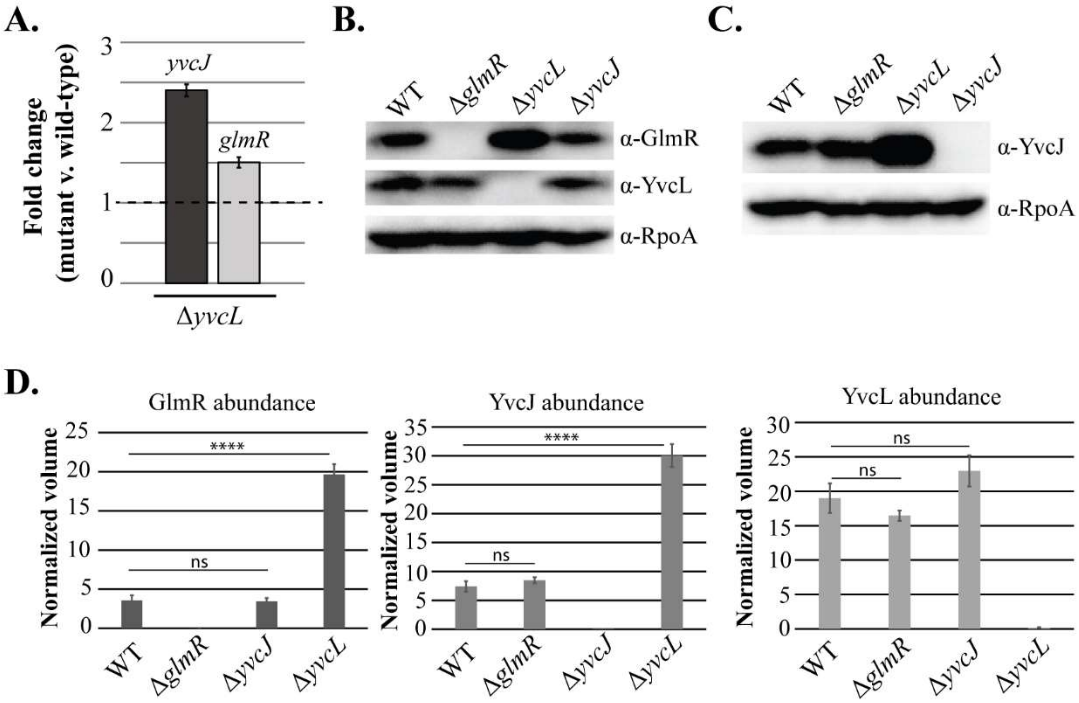

S9 Fig. Abundance of GlmR and YvcJ is elevated in the absence of YvcL. A. Expression of *yvcJ* and *glmR* genes was measured using RT-qPCR on RNA extracted from exponentially growing wild-type (WT) and Δ*yvcL* strains in MH broth. Strains used: wild-type, OG1; Δ*yvcL*, DDJ260. **B.** Bacteria were grown to exponential phase in MH broth. Whole-cell lysates were subjected to SDS-PAGE and protein expression assessed via immunoblot analysis for GlmR, YvcL, or RpoA (loading control). **C.** Bacteria were grown to exponential phase in MH broth. Whole-cell lysates were subjected to SDS-PAGE and protein expression assessed via immunoblot analysis for YvcJ or RpoA (loading control). **D.** Quantification of abundance of each protein, from panels B and C, normalized to total protein in each lane using four biological replicates. ****, $p < 0.0001$ determined via *t* test. *ns*, $p > 0.05$. Strains used were: wild-type, OG1; Δ*glmR*, DDJ245, Δ*yvcL*, DDJ260; Δ*yvcJ*, DDJ326.
