# Supplementary material for: Reciprocal regulation of enterococcal cephalosporin resistance by products of the autoregulated yvcJ-glmR-yvcL operon enhances fitness during cephalosporin exposure
Source: PLoS Genet. 2024 Mar 21;20(3):e1011215. doi: 10.1371/journal.pgen.1011215 (PMC10986989; doi:10.1371/journal.pgen.1011215)
Supplement: S10 Fig — Whole-cell lysates from E. faecalis cells grown exponentially in MH broth (supplemented with erythromycin for maintenance of plasmids and +/- 5 mM NaNO3) were subjected to immunoblot analysis. Strains and plasmids used were: wild-type (WT), OG1; ΔglmR, DDJ245; ΔyvcL, DDJ260; ΔyvcJ, DDJ326; vector, pJLL286; PnisA-glmR, pDDJ262; PnisA-yvcL, pDDJ271; PnisA-yvcJ, pDDJ269. (PDF) [file pgen.1011215.s019.pdf]

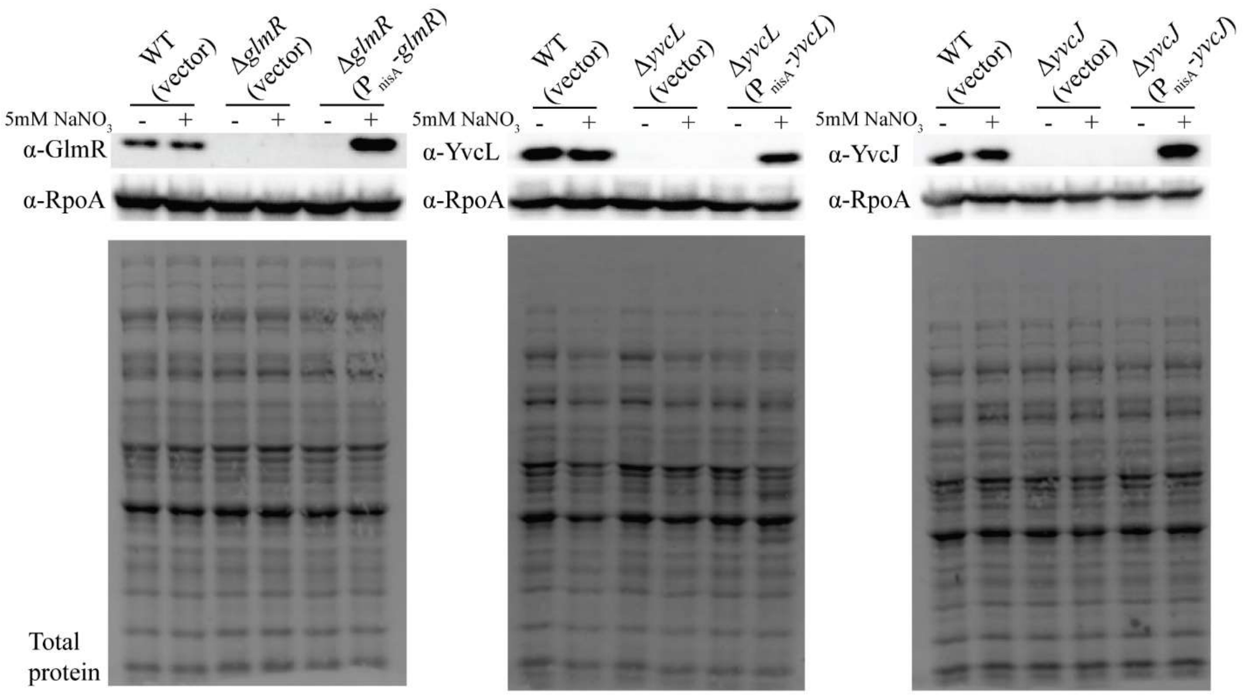

S10 Fig. Abundance of YvcJ and YvcL upon induction of expression using 5 mM nitrate. Whole-cell lysates from *E. faecalis* cells grown exponentially in MH broth (supplemented with erythromycin for maintenance of plasmids and +/- 5 mM $NaNO_3$) were subjected to immunoblot analysis. Strains and plasmids used were: wild-type (WT), OG1; Δ*glmR*, DDJ245; Δ*yvcL*, DDJ260; Δ*yvcJ*, DDJ326; vector, pJLL286; P_{nisA}-*glmR*, pDDJ262; P_{nisA}-*yvcL*, pDDJ271; P_{nisA}-*yvcJ*, pDDJ269.
