# Supplementary material for: Reciprocal regulation of enterococcal cephalosporin resistance by products of the autoregulated yvcJ-glmR-yvcL operon enhances fitness during cephalosporin exposure
Source: PLoS Genet. 2024 Mar 21;20(3):e1011215. doi: 10.1371/journal.pgen.1011215 (PMC10986989; doi:10.1371/journal.pgen.1011215)
Supplement: S11 Fig — Whole-cell lysates from E. faecalis cells grown exponentially in MH broth were subjected to immunoblot analysis. Quantification of abundance of MurAA normalized to total protein in each lane was done from three biological replicates. Wild-type (WT), OG1; Δ(yvcJ-glmR), DDJ338. RpoA is a loading control. (PDF) [file pgen.1011215.s020.pdf]

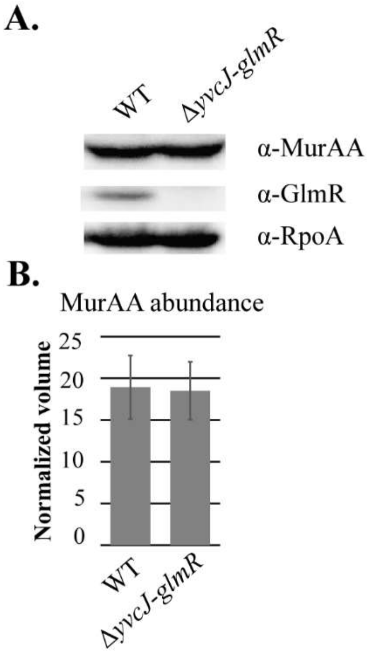

S11 Fig. **MurAA abundance is not altered in the Δ(*yvcJ-glmR*) double mutant.**
Whole-cell lysates from *E. faecalis* cells grown exponentially in MH broth were subjected to immunoblot analysis. Quantification of abundance of MurAA normalized to total protein in each lane was done from three biological replicates. Wild-type (WT), OG1; Δ(*yvcJ-glmR*), DDJ338. RpoA is a loading control.
