# Supplementary material for: Reciprocal regulation of enterococcal cephalosporin resistance by products of the autoregulated yvcJ-glmR-yvcL operon enhances fitness during cephalosporin exposure
Source: PLoS Genet. 2024 Mar 21;20(3):e1011215. doi: 10.1371/journal.pgen.1011215 (PMC10986989; doi:10.1371/journal.pgen.1011215)
Supplement: S12 Fig — Sequences of the proteins (E. faecalis GlmR accession AEA93188; B. subtilis GlmR accession NP_391356) were aligned and compared using a Web version of ClustalOmega. (*) signifies conserved residues. (PDF) [file pgen.1011215.s021.pdf]

```
E. faecalis MKTYRIRKPKIVVVGGGTGLPVILKSLRNQSVDITAVVTVADDGGSSGELRSSINNMTPP 60
B. subtilis ----MGQKPKIAIFGGGTGLSVLLRGLKHKPVDITAIVTVADDGGSSGRLRNEL-KIPPP 55
                  :****.:.****** *:*:.*::: *****:***********.**..: :: **

E. faecalis GDLRNVLVALSDMPQLYEDIFQYRFDKSDSHFANHAIGNLIIAAVSEMRGSTYEAIQLLS 120
B. subtilis GDIRNVLAALSDVEPLVEDLFQHRFNKGN-DLTGHSLGNLILAAMTNITGDFFHAVTEMS 114
            **:****.****:  * **:**:**:*.: .::.*::****:**:::: *. :.*:  :*

E. faecalis KMMHVDGRIYPSSERPLTLHAVFKDGSVAVGESKIALDRKTIDHVFVTNTHGEEQPRAAR 180
B. subtilis KVLNVRGKVLPAANASVVLHAEMEDGRVVSGESTIPEYGQRIKRVFLTPEQIDPL----P 170
            *:::* *:: *:::  :.*** ::** *. ***.*    : *.:**:*  : :

E. faecalis KVVKAIEEADMVVLGPGSLFTSILPNLVITEIGEAIKQTAAEVVYICNIMTQKGETEHFT 240
B. subtilis ETIDVIREADLIIIGPGSLYTSILPNLLVPKIGEEVIKAPAKKVYICNVMTQPGETLHYT 230
            :.:..*.***::::*****:*******:: :*** : :: *: *****:*** *** *:*

E. faecalis DADHVRVLNEHLQAQFVDTVLVNTEKVPENYMDPEIYDEYLVQVKHDFQGLREEGCRVIS 300
B. subtilis AADHVKALNQHMGCGFIDTILVNSEDIPDEIKRKYEM-ESARPVDFDIEELKAMGLEVIR 289
             ****:.**:*: . *:**:***:*.:*::        *    *..*:: *:  * .**

E. faecalis TDFLELRDGGVFHDGEKVVEELFRIVFGTKY 331
B. subtilis DQIVTYKNDVIRHDTHKVASLLVDLLKE--- 317
             :::  ::. : ** .**.. *. ::
```

**S12 Fig. Alignment of GlmR proteins from *E. faecalis* and *B. subtilis*.** Sequences of the proteins (*E. faecalis* GlmR accession AEA93188; *B. subtilis* GlmR accession NP_391356) were aligned and compared using a Web version of ClustalOmega. (*) signifies conserved residues.
