# Supplementary material for: Reciprocal regulation of enterococcal cephalosporin resistance by products of the autoregulated yvcJ-glmR-yvcL operon enhances fitness during cephalosporin exposure
Source: PLoS Genet. 2024 Mar 21;20(3):e1011215. doi: 10.1371/journal.pgen.1011215 (PMC10986989; doi:10.1371/journal.pgen.1011215)
Supplement: S13 Fig — Whole-cell lysates from E. faecalis cells grown exponentially in MH broth were subjected to immunoblot analysis. Quantification of abundance of YvcJ and GlmR normalized to total protein in each lane was done from two biological replicates. Strains used were: WT, OG1; ΔyvcJ, DDJ326; yvcJK18A, DDJ446. RpoA is the loading control. (PDF) [file pgen.1011215.s022.pdf]

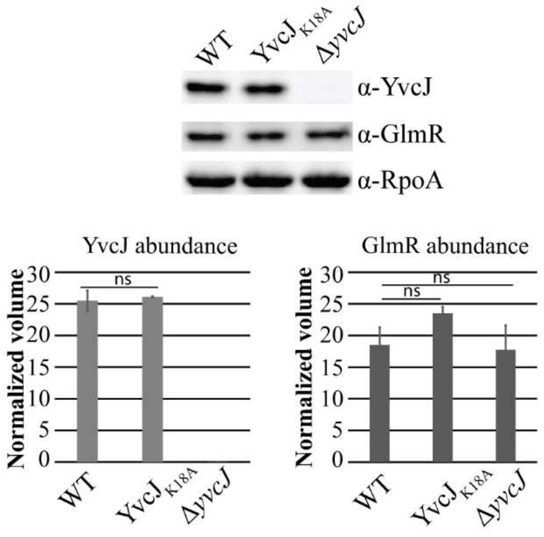


**S13 Fig. YvcJ$_{\mathbf{K18A}}$ and GlmR expression is unaltered in the *yvcJ*$_{\mathbf{K18A}}$ mutant**. Whole-cell lysates from *E. faecalis* cells grown exponentially in MH broth were subjected to immunoblot analysis. Quantification of abundance of YvcJ and GlmR normalized to total protein in each lane was done from two biological replicates. Strains used were: WT, OG1; Δ*yvcJ*, DDJ326; *yvcJ*$_{\text{K18A}}$, DDJ446. RpoA is the loading control.
