# Supplementary figures and images for: Reciprocal regulation of enterococcal cephalosporin resistance by products of the autoregulated yvcJ-glmR-yvcL operon enhances fitness during cephalosporin exposure
Source: PLoS Genet. 2024 Mar 21;20(3):e1011215. doi: 10.1371/journal.pgen.1011215 (PMC10986989; doi:10.1371/journal.pgen.1011215)

**S14 Fig.** Images used for quantitation in Fig 3

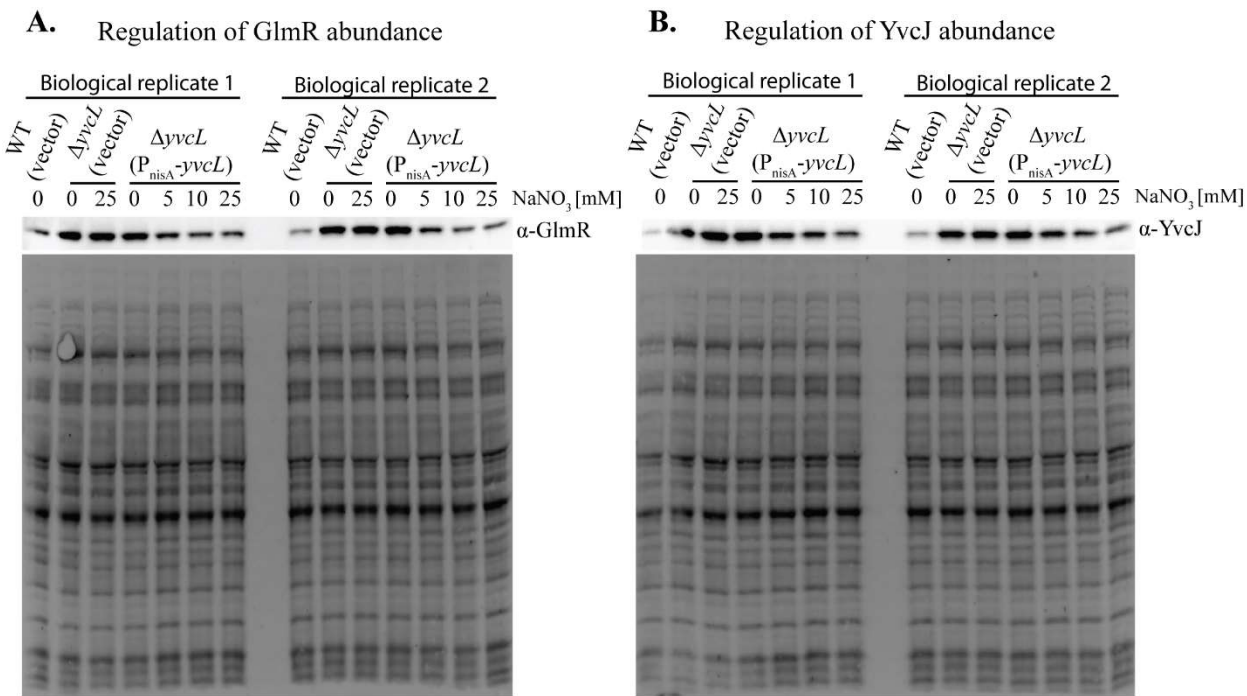

Supplement: S14 Fig — (PDF) [file pgen.1011215.s023.pdf]

**S15 Fig.** Images used for quantitation in S9 Fig.

**B.**

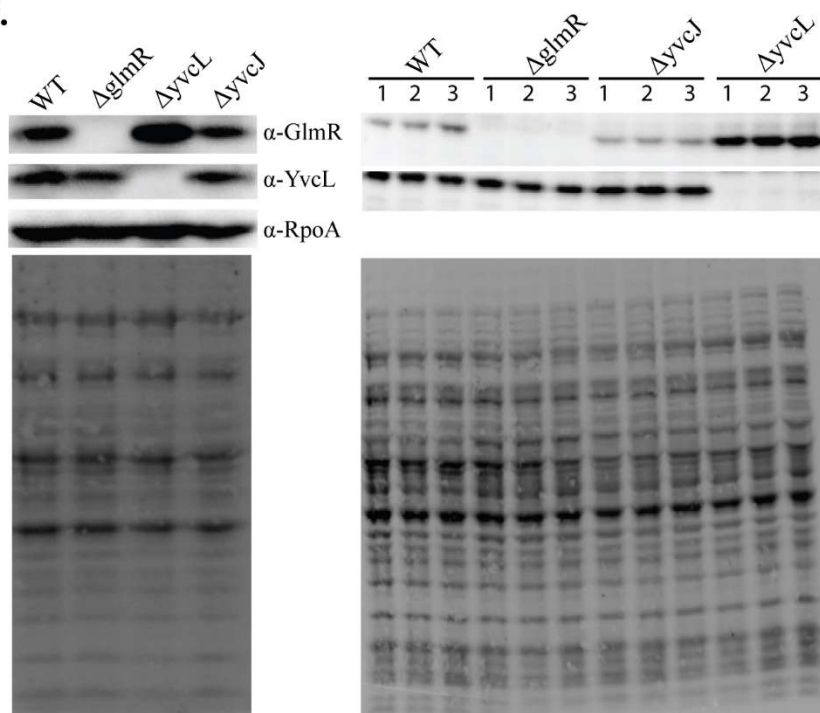

**C.**

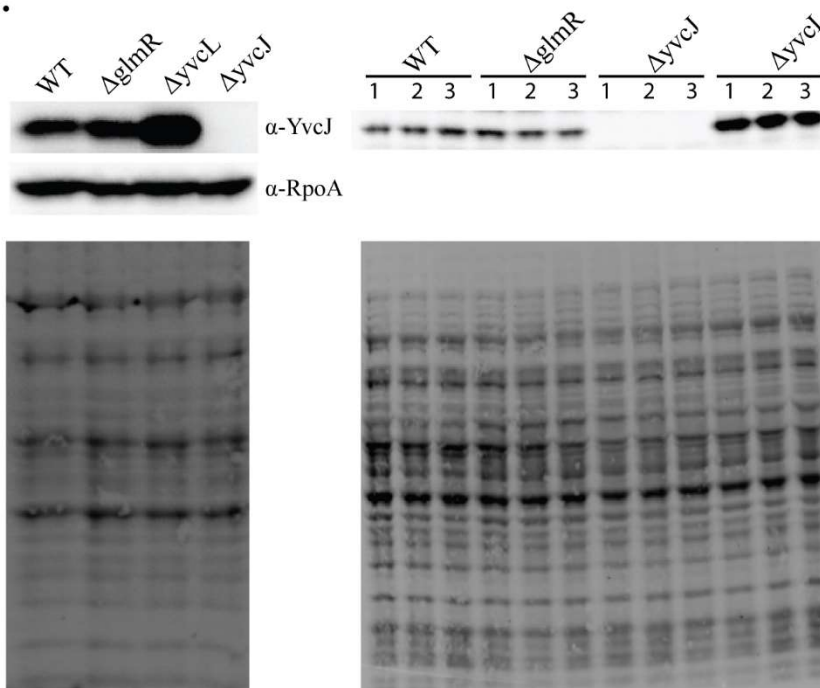

Supplement: S15 Fig — (PDF) [file pgen.1011215.s024.pdf]

**S16 Fig.** Images used for quantitation in S11 Fig

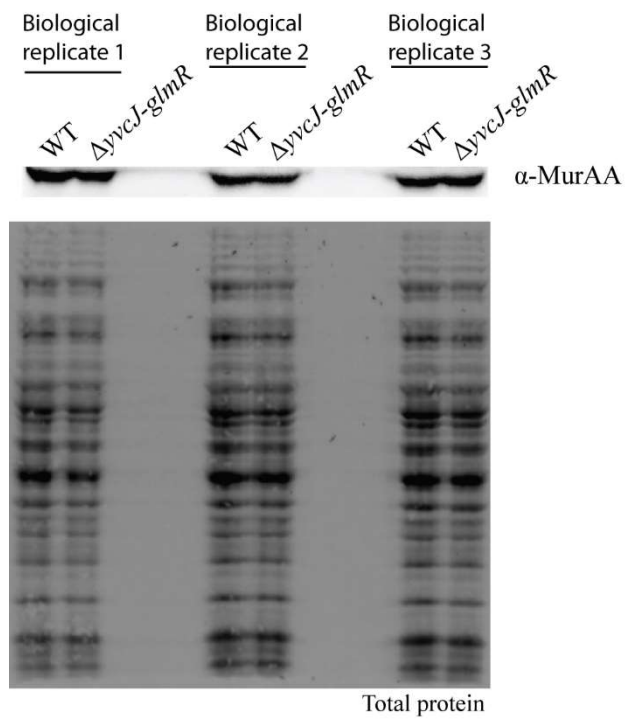

Supplement: S16 Fig — (PDF) [file pgen.1011215.s025.pdf]

**S17 Fig.** Images used for quantitation in S13 Fig

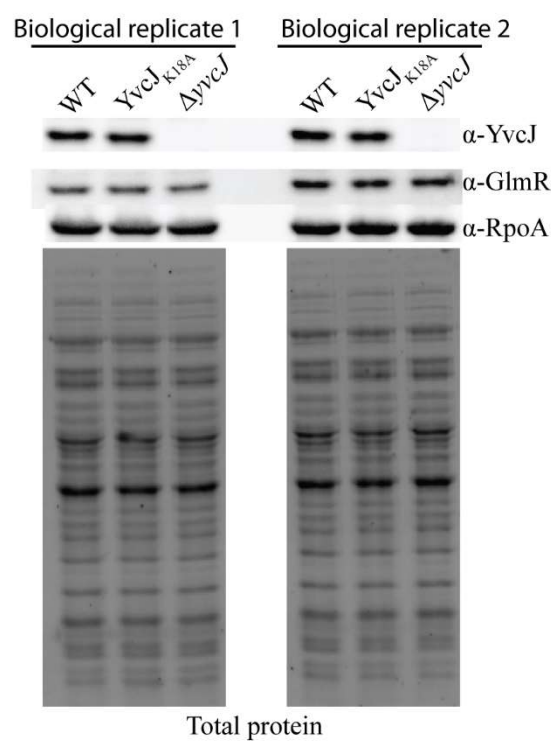

Supplement: S17 Fig — (PDF) [file pgen.1011215.s026.pdf]
